# Ethnozoology in Brazil: current status and perspectives

**DOI:** 10.1186/1746-4269-7-22

**Published:** 2011-07-18

**Authors:** Rômulo RN Alves, Wedson MS Souto

**Affiliations:** 1 Departamento de Biologia, Universidade Estadual da Paraíba, Av. das Baraúnas, 351/Campus Universitário, Bodocongó, 58109-753, Campina Grande-PB, Brasil; 2 Programa de Pós-Graduação em Ciências Biológicas (Zoologia), Departamento de Sistemática e Ecologia, Universidade Federal da Paraíba, 58059-970 João Pessoa, PB, Brazil

## Abstract

Ancient connections between animals and human are seen in cultures throughout the world in multiple forms of interaction with the local fauna that form the core of Ethnozoology. Historically, ethnozoological publications grew out of studies undertaken in academic areas such as zoology, human ecology, sociology and anthropology - reflecting the interdisciplinary character of this discipline. The rich fauna and cultural diversity found in Brazil, with many different species of animals being used for an extremely wide diversity of purposes by Amerindian societies (as well as the descendents of the original European colonists and African slaves), presents an excellent backdrop for examining the relationships that exist between humans and other animals. This work presents a historical view of ethnozoological research in Brazil and examines its evolution, tendencies, and future perspectives. In summary, literature researches indicated that ethnozoology experienced significant advances in recent years in Brazil, although from a qualitative point of view improvement is still needed in terms of methodological procedures, taxonomic precision, and the use of quantitative techniques. A wide range of methodologies and theories are available in different areas of learning that can be put to good use in ethnozoological approaches if the right questions are asked. The challenges to studying ethnozoology in Brazil are not insignificant, and the tendencies described in the present study may aid in defining research strategies that will maintain the quantitative growth observed in the recent years but likewise foster needed qualitative improvements.

## Introduction

There have been extremely close connections of dependence and co-dependence between humans and animals throughout history [1-7]. Research suggests that humans evolved from a vegetarian lifestyle to the one including meat in their diets around 2.5 million years ago (at the dawn of the genus *Homo*) [8,9], though just how much of the prehistoric diet included animals is difficult to tell from archeological evidence [10]. Up until around 12,000 years ago, humans derived food and raw materials from wild animals and plants [11]. Other evidence of ancient human-animal relationships can be seen in rock paintings that depict wild animals such as bison, horses and deer with human figures hunting them. This sort of evidence corroborates the observation of Marques [12] that human-animal interactions have constituted basic connections in all societies throughout history.

The variety of interactions (both past and present) that human cultures maintain with animals is the subject matter of Ethnozoology, a science that has its roots as deep within the past as the first relationships between humans and other animals. According to Sax [13], human attitudes towards animals probably evolved long before our first attempts to portray them artistically or examine them scientifically. In this sense, it has been speculated that the origin of ethnozoology coincides with the appearance of humans as a species or, perhaps more correctly, with the first contacts between our species and other animals [14]. This view of ethnozoology assumes that these interactions are an integral part of human culture and society.

The rich fauna and cultural diversity found in Brazil, with many different species of animals being used for an extremely wide diversity of purposes by Amerindian societies (as well as the descendents of the original European colonists and African slaves), presents an excellent backdrop for examining the relationships that exist between humans and other animals. The first records and contributions to ethnozoology were produced by early naturalists and explorers who demonstrated interest in the fauna as well as the zoological knowledge of native residents. These naturalists generally compiled lists of native animals together with their regional and scientific names and descriptions of their uses [15]. Nevertheless, the scientific research in the area has been intensifying in recent years, and Brazil is currently one of the most important sources of scientific production in this area.

The history of ethnozoology cannot be separated from the history of zoology, and the first records and contributions to this discipline were produced by naturalists and explorers. Historically, ethnozoological publications grew out of studies undertaken in academic areas such as zoology, human ecology, sociology and anthropology - reflecting the interdisciplinary character of ethnozoology. This review presents an historical view of ethnozoological research in Brazil and examines its evolution, tendencies, and future perspectives.

## Procedures

In examining the development and tendencies of Ethnozoology in Brazil, we analyzed papers published on this theme through March/2011. Only texts that had been published in scientific periodicals, books, or book chapters that considered human/faunal relationships were considered. Searches were made for articles available through international online databases such as Web of Science, Scopus, and Google Scholar as well as specific journal web sites. We used the following search key words: Ethnozoology, Ethnoentomology, Ethnoichthyology, Historical ethnozoology, Cynegetic activities, Ethnocarcinology, Ethnoornithology, Ethnotaxonomy, Ethnomastozoology, Ethnoherpetology, Ethnomalacology, Animal use and Zootherapy. It is important to note that a number of papers could be classified into more than one category, but for purposes of this revision we considered only the principal theme of the work in deciding its category (e.g. a publication focused on the medicinal uses of reptiles was considered under the heading of zootherapy, and not ethnoherpetology. We recorded the location where the works were published, which allowed to identify their distribution according to biomes and regions where the studies were performed.

## The first works

The first paper published in Brazil with a strict ethnozoological focus appeared in 1939 and described the popular zoological vocabulary used by Brazilian natives [16]. It must be noted, however, that when the first naturalists, colonists, and Jesuits arrived in the country in the 16th century they encountered an abundant, diversified and strange fauna waiting to be documented. According to Ribeiro [17], the discovery of a whole new world in the Americas generated tremendous curiosity among Europeans about the new and different plants and animals that thrived in those lands. In the centuries that followed these first contacts, explorers, chroniclers and naturalists from many disciplines and many parts of Europe set out to describe this exotic cultural universe and the fantastic and unique natural world.

These historical documents provided descriptions of the local fauna and described the hunting techniques employed by local natives in embryonic ethnozoological approaches. According to Papavero [18], the indigenous tribes, notably those who spoke the Tupi language, acted as the first professors of natural history in Brazil, transmitting their detailed knowledge of the fauna and flora to the Jesuits, who were, in this area at least, their students. Based on the information provided by these native tribes, the members of this religious order recorded the first lists and vocabularies of the local fauna. Among these missionaries were José de Anchieta, Gaspar Affonso, Francisco Soares and, especially, Leonardo de Valle who listed nothing less than 351 Tupi names for different animals (in about 1585) - a valuable linguistic and ethnozoological document that was only recently published. Little by little, expeditions through South America revealed an extremely rich fauna composed of animals of rare beauty, such as parrots and macaws (which led to Brazil being called for a certain time the "Land of Parrots"), as well as strange creatures that were very different from any previously known to Europeans. These findings stimulated the naturalists of that time to formulate various theories about the geographical distribution of species in the world [18].

Given that naturalists have been recording ethnozoological knowledge since colonial times, one could consider the roots of ethnozoological in Brazil as dating from the 16^th ^century - so that the history of ethnozoology in Brazil blends into the history of zoology itself. In truth, it can be said that ethnozoology is old in practice but young in theory, for the discipline is not as modern as it might first appear, with roots going back to the earliest relationships between animals and humans. A number of initiatives began to appear to recuperate zoological data from colonial period documents - an academic area that can be called Historical Ethnozoology. Nelson Papavero (at the University of São Paolo), Dante Luiz Martins Teixeira (Federal University of Rio de Janeiro), and Hitoshi Nomura (University of São Paolo) have published a series of papers on this theme in Brazil [eg. [19-25]]

## Ethnozoological research in Brazil

**Figure 1:**
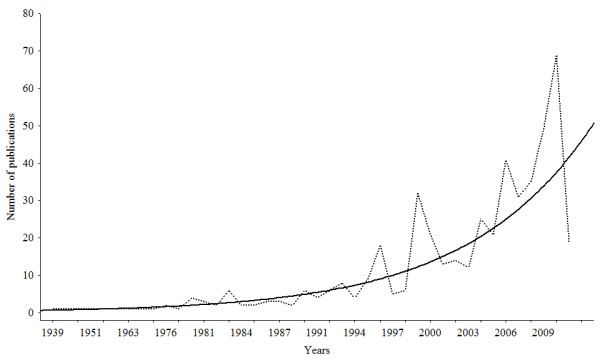
**Temporal distribution of Ethnozoological research in Brazil**. Crude data (dotted line) and data adjusted to an exponential growth curve.

If on one hand it can be said that ethnozoological documentation dates to the 16^th ^century, scientific production in this area only began to gain form in Brazil near the beginning of the 21^st ^century (Figure 1). In analyzing the distribution of publications (scientific periodicals, books and book chapters) over the years we noted that a large majority of the research on this theme (350 (73.3%) of 487 works) were published within just the last ten years (coinciding with an increase in published works in the many areas of ethnosciences in that country). A review undertaken by Oliveira *et al*.[26] in the field of ethnobotany, for example, revealed that the numbers of publications in scientific journals had experienced an expressive expansion in the last decade.

The notable concentration of ethnozoological publications in recent years in Brazil is consistent with the historical development of this discipline. The academic development of ethnobiology in this country is only very recent, and greater numbers of publications in recent years would therefore be expected. A total of 487 works were published up until July 2011 (Figure 1). Starting with the first ethnozoolology publication in 1939, the following years were characterized by small productions (a maximum of six publications/year). In the 1990's publications begin to appear in greater numbers, but only in the 21^st ^century did journal production really reach expressive numbers. Likewise the diversity of themes examined in ethnozoological research became more numerous and diversified during the Brazilian Ethnobiology and Ethnoecology Symposiums, the National Zoology Congresses, and the Brazilian Ecology Congresses held in recent years; it is hoped that this growth will soon be reflected in increased numbers of publications.

**Figure 2:**
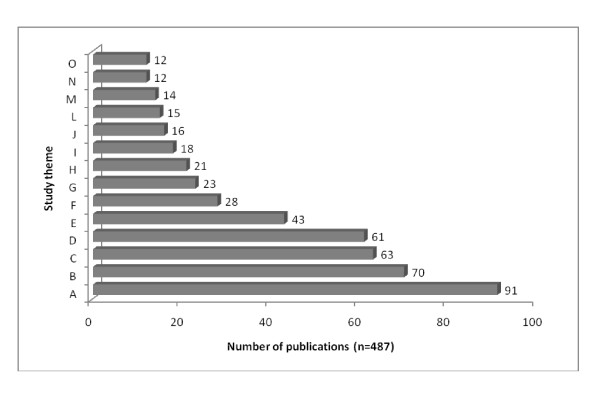
**Distribution of Ethnozoology research in the Brazil according to the study theme**. A - Zootherapy, B - Others, C - Ethnoentomology, D - Ethnoichthyology, E - Historical ethnozoology, F - Cynegetic activities, G - Ethnocarcinology, H - Ethnoornithology, I - Education and management, J - Ethnotaxonomy, L - Magic-religious purposes and cultural symbolisms, M - Ethnomastozoology, N - Ethnomalacology, O - Ethnoherpetology.

Figure 2 lists the themes of ethnozoological publications discussed in the present revision. The subjects considered in these publications can be divided into 13 categories, with the specific themes most frequently treated being: zootherapy - the use of animals and their sub-products in folk medicine (17.86% of the titles), ethnoentomology (12.94%), ethnoichthyology (12.32%), historical ethnozoology (8.83%), cynegetic activities (hunting activities) (5.75%), ethnocarcinology (4.72% each), ethnoornithology (4.11%), ethnotaxonomy (3.08%), education and management (3.7%), the use of animals for magic-religious purposes and cultural symbolisms (3.08%), ethnomastozoology (2.87%), ethnoherpetology (2.46%), and ethnomalacology (2.26%). Any work that did not fit well into the above mentioned categories was classified as "others" (1.02%).

One of the principal reasons that Ethnozoology is still only poorly studied in Brazil is related to legal problems associated with the use of wild animals. Hunting is completely prohibited in the country, and this is known to anyone who sells or uses animal products (making full cooperation with researchers much more difficult). The legal implications of the protection of the local fauna will in turn influence the choice of topics for ethnozoolology studies. The result is that themes such as ethnoichthyology and ethnoentomology represent a significant percentage of the publications - a situation associated with the importance of these faunal groups, but also with the fact that these animals (fish and insects) can generally be used or sold without excessive legal restrictions and this is one reason why there are more studies on this subject. In the case of ethnoichthyology, it is noted that even fishers' behavior and fisheries management have been the object of many studies. The human populations that harvest these resources generally feel more secure about sharing information about their activities. On the other hand, researchers who might wish to study the hunting of wild animals - a very common practice in Brazil in spite of its notorious illegality - will have to overcome considerably more suspicion and reluctance on the part of their informants.

**Figure 3:**
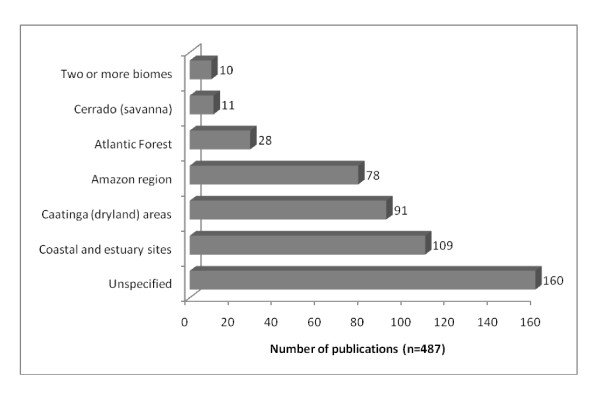
**Distribution of Ethnozoological research in Brazil by biome**.

The focus of ethnozoology publications varies according to the region in which they are developed, as would be expected. The realities of each region, including its cultural diversity and the diverse types of ecosystems that occur there, will strongly influence research directions. Studies dealing with fishing resources (fish, crustaceans and mollusks) are more frequently undertaken in coastal areas, for example, while most of the published papers from the Amazon region have dealt with cynegetic animals and the use of the local fauna by indigenous groups. The environments in which the largest numbers of research projects were undertaken were: coastal and estuary sites (22.38%, n = 109 studies), Caatinga (dryland) areas (18.69%, n = 91), the Amazon region (16.02%, n = 78), and the Atlantic Forest (5.75%, n = 28). Only eleven studies were produced in the Cerrado (savanna) biome (2.26%), and no studies were published focusing on the Pantanal seasonal wetlands. A few projects (n = 10, 2.05%) were undertaken in two or more biomes; many were general studies (32.85%, n = 160) and not restricted to specific biomes (Table 1, Figure 3).

**Table 1:** Ethnozoological studies published in Brazil by theme, region and biome.

STUDY THEMES	BIOMES							REGIONS*						
	
	Amazon region	Caatinga (dryland) areas	Cerrado (savanna)	Atlantic Forest	Coastal and estuary sites	Two or more biomes	Unspecified	N	NE	N-NE	S	SE	CO	UN
**Cynegetic activities**	[29-41]	[6,42-49]		[50-52]	[53]	[54]	[55]	[29-32,34-37,39-41]	[6,42-49,52]			[50,51,53]	[38]	[33,54,55]
**Education and management**	[56-58]	[59]		[60-62]	[63-69]		[70-73]	[56-58]	[59-61,65-67]			[62,64,68-70]		[63,71-73]
**Ethnocarcinology**	[74]	[75-77]			[78-95]		[96]	[74]	[75-81,83-88,91,92,94]			[89,90,93,95]		[82,96]
**Ethnoentomology**	[97-106]	[107-130]	[131,132]	[133-136]			[137-159]	[97,98,100-106]	[107-130,134,136,141,142,146,158]		[133,159]	[135,152]	[99,131,132]	[137-140,143-145,147-151,153-157]
**Ethnoherpetology**	[160,161]	[162-166]					[167-171]	[160,161]	[162-166]					[167-171]
**Ethnoichthyology**	[172-179]	[180-183]	[184]	[185-188]	[189-222]	[223]	[224-231]	[172,173,175,177-179]	[176,180-183,185,194-198,202,203,205,210,218,219]	[211,212,221]	[199,229]	[186-191,193,200,201,204,206-209,213-215,220,225]	[174,184]	[192,216,217,222-224,226-228,230,231]
**Ethnomalacology**	[232]		[233]	[234]	[235-240]	[241]	[242]	[232]	[234-241]				[233]	[242]
**Ethnomastozoology**	[243]	[244,245]	[246]	[247]	[248-250]		[251-256]	[243]	[244,245,247]		[248,254,255]	[250,252]	[246]	[249,251,253,256]
**Ethnoornithology**	[257]	[258-262]	[263,264]	[265,266]	[267]	[268]	[269-276]	[257]	[258-262,265-268,271,272,276]			[263,264,274]		[269,270,273,275]
**Ethnotaxonomy**	[277,278]	[279,280]			[281-289]	[290]	[291]	[277,278]	[279,280,282-287,289,291]			[281,288]		[290]
**Historical ethnozoology**	[21,24,292-306]			[307]			[18,20,22,23,308-328]	[21,24,292-306]	[22]			[307,315,316,318,320]	[319]	[18,20,23,308-314,317,321-328]
**Magic-religious purposes and cultural symbolisms**	[329-331]	[332-335]					[336-343]	[330,331]	[332-335,341]	[329]				[336-340,342,343]
**Zootherapy**	[344-350]	[351-378]	[379]	[380-382]	[383-388]	[389-392]	[393-431]	[344-346,349,350]	[348,351-378,380-383,388,389,403-405,409,411,422,430]	[384-386,390,391]		[387]	[379]	[347,392-402,406-408,410,412-421,423,424,426-429,431]
**Others**	[432-440]	[441-444]	[445-447]	[448-453]	[454-477]	[478,479]	[14,16,431,480-506]	[432-440,467,479,483,506]	[442,443,452-457,460-466,468,471,473-476,486,491,492,505]	[441,494]	[477,480,481]	[447-451,458,459,469,470,472,478,484]	[445,446]	[14,16,444,482,485,487-490,493,495-504]

N - Northern region, NE - Northeastern region, N-NE - Northern and Northeastern regions, S - South region, SE - Southern region, CO - Central-western region, UN - Unspecified

**Figure 4:**
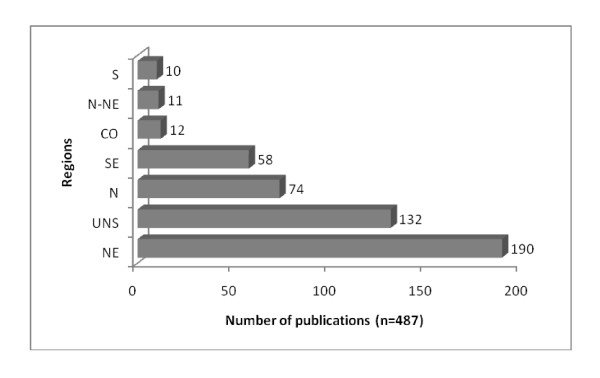
**Distribution of Ethnozoological research in Brazil by region**. UNS = Unspecified.*Legend: N - Northern region, NE - Northeastern region, N-NE - Northern and Northeastern regions, S - South region, SE - Southern region, CO - Central-western region, UNS = Unspecified.

In spite of the quantitative increase of published reports in Brazil, there are still regional imbalances in terms of ethnozoological research and associated scientific production - with research being concentrated in the northeastern region of that country (39%, n = 190) (especially in the states of Bahia and Paraíba). Many of these studies were undertaken in the northern (15.2%, n = 74) and southeastern (11.9%, n = 58) regions of Brazil. In contrast, relatively few ethnozoological studies have been produced focusing on areas in the central-western and southern regions of the country (twelve (2.4%) and ten (2.0%) studies respectively). Eleven studies have been published concerning work undertaken in cities in northern and northeastern Brazil, while 27.1% (n = 132) did not foci on any specific region Figure 4).

The recent quantitative advances in ethnozoological publications were in large part due to the work of new researchers employed in research and teaching positions throughout Brazil who (together with the pioneer researchers) have greatly contributed to the growth of this area. Some of the articles published (n = 31, 6.3%) include the participation of foreign researchers, showing the existence of international links and interactions between researchers from Brazil and others countries. It must be pointed out, however, that the numbers of researchers directly involved with ethnozoological inquires in Brazil are still very small, although many zoologists and ecologists have undertaken research programs in this area even though ethnozoology is not their principal line of research. Another important aspect related to recent advances in ethnozoology is the fact that this subject is now offered in many graduate courses, even in largely specific departments such as Zoology and Ecology (e.g. the State University of Paraiba, and the State University of Feira de Santana). As such, there have been significant increases in the availability of advisors as well as in the numbers of graduate courses on this theme- which have contributed to the recent advances in ethnozoological studies in Brazil and reinforced the growth of this field.

Ethnozoological research papers have appeared in many different national (66.9%, n = 326) and international (33.%, n = 161) publications. Among the texts identified, most have appeared in scientific periodicals. Although these journal articles are the most frequent type of ethnozoological publication, there are currently no specialized ethnozoological journals published in Brazil (and even on a global scale there are relatively few journals focused on ethnobiology). As such, ethnozoological articles have been published in journals covering many different areas, such as traditional medicine, conservation, ethnography, conservation and management, among others. Although the multidisciplinary nature of ethnozoology permits different types of articles to be published in different journals (which has been an important factor stimulating the growth of scientific production on these themes), the results of our present study reinforce the necessity of establishing more journals with specific ethnobiological focuses that can accept texts in both ethnozoology and ethnobotany.

Brazil stands out as one of the world's leading producers of ethnozoological studies. These quantitative advances indicate that the country will continue to have an important role in ethnozoological research, and this same tendency has been observed for ethnobotany [26] - which places this country in the global vanguard of ethnobiological inquires. In spite of this optimistic outlook, however, it is important to note that human resources with specializations in ethnozoology are still relatively scarce, and research centers in this area are restricted to just a few states in the country. On the other hand, interactions between ethnozoologists, zoologists and ecologists have been increasing and will certainly generate more publications and improvements in research quality.

In spite of the quantitative growth of ethnozoological research, there is a clear need for qualitative improvements in the publications generated. Many of the journal articles have had strongly descriptive natures, based simply on lists of species (which are often taxonomically incorrect or are restricted to just the common names of the animals). There is a necessity for planning and preparing studies with greater scientific rigor; for studies addressing specific questions and hypotheses; as well as theoretical and methodological advances that will help consolidate ethnozoology. In their review of ethnobotany in Brazil, Oliveira *et al*. [26] noted the tendency to incorporate hypotheses as well as discussions and critical analyses of methodologies, as well as a movement towards focusing on the resolution of practical questions - tendencies that should likewise guide ethnozoological and ethnobiological researchers. The document "Intellectual Imperatives in Ethnobiology" [27], an international guideline to do ethnobiological research, makes it very clear that research projects in ethnobiology should be guided by hypotheses, that appropriate collaborators must be included to assure the use of rigorous methodologies inspired by different but related disciplines, and that statistical analyses and rigorous and appropriate mathematical models must be used to guide data collection and analysis [27].

As was noted by Oliveira [26], a number of important events have contributed to the development of the ethnosciences (including ethnozoology) in Brazil, including: the publication of the first edition of "Suma Etnológica Brasileira" [28]; the success of the I International Congress of Ethnobiology in 1988 in Belém, Pará State (during which the International Ethnobiology Society [ISE] was founded); the foundation of the Brazilian Society of Ethnobiology and Ethnoecology (SBEE) during the I Brazilian Symposium of Ethnobiology and Ethnoecology held in 1996; as well as numerous other national, regional and state-level symposia of ethnobiology and ethnoecology that have taken place in recent years. More recently (in February/2010), the I Brazilian Symposium of Ethnozoology was held during the XXVIII Brazilian Congress of Zoology in Belém, Pará State; and in November/2010 the VIII Brazilian Symposium of Ethnobiology and Ethnoecology and the II Latin-American Congress of Ethnobiology took place in Recife, Pernambuco State. As was noted by Oliveira *et al*. [26], the SBEE has assumed an important role in the promotion of different forums for debate in which professionals from the area have been able to discuss the perspectives, limitations, conceptual and theoretical questions, theories, and methodologies, as well as the political and social implications of research in this area. The incorporation of ethnozoology into graduate programs has likewise made important contributions to this process. The challenges that the ethnosciences must face in the coming years include the amplification of graduate programs in regions and biomes that have been as yet little studied, as well as the continued thematic diversification of the field - which will help Brazilian ethnozoology consolidate itself as a modern and multidisciplinary science aligned with international research standards.

Ethnozoology currently confronts a number of challenges, and some of the most urgent items include the establishment of efficient dialogs between different academic areas that interface with ethnozoology; qualitative improvements in research techniques; greater scientific rigor; consolidation of undergraduate and graduate courses; exchanges of experiences in relation to the results produced and the methodologies utilized; and the development of monitoring programs based on sound research into the conservation and sustainable use of natural resources.

One of the main characteristics of human knowledge is its dynamism [26]. Reformulations of objectives, methodologies and theories occur in all of the sciences from time to time - and ethnozoology will not be different in this respect. The fact that ethnozoology has been the target of many recent criticisms has helped transform it into an area of scientific study bursting with new ideas and different reflections. As was noted by Oliveira [26], at a time when the world is debating so many polemic themes concerning the benefits and dangers of scientific/technological advances, the ethnosciences are discussing the possibility of linking scientific research to human priorities (especially to aid traditional populations and societies that have been historically excluded), the urgent necessities of conservation, and the more parsimonious use of natural resources.

In summary, literature researches indicated that ethnozoology has experienced significant advances in recent years in Brazil - although this discipline is still in the process of developing a sound theoretical base and unified methodological programs. A wide range of methodologies and theories have arisen in different areas of learning that can be put to good use if the right questions are asked using ethnozoological approaches.

The dynamism of this discipline in Brazil can be confirmed in the quantitative and qualitative growth of research papers published in scientific journals and discussed at related national events. More proof of the approaching maturity of this discipline can be seen in the numbers of internationally respected Brazilian ethnozoologists who are directly involved in the progress seen in their fields, and the participation of a many Brazilian researchers on editorial commissions and as consultants in renowned periodicals. From a qualitative point of view, however, improvement is still needed in terms of methodological procedures, taxonomic precision, and the use of quantitative techniques. The challenges to studying ethnozoology in Brazil are not small, and the tendencies described in the present study may aid in defining research strategies that will maintain the quantitative growth observed in the recent years but likewise foster needed qualitative improvements.

## Competing interests

The authors declare that they have no competing interests.

## Authors' contributions

RRNA and WMSS worked in the bibliographical classification, conception and the article final composition. The authors read and approved the final manuscript.

## References

[B1] Pataca EM A Ilha do Marajó na Viagem Philosophica (1783-1792) de Alexandre Rodrigues Ferreira Boletim do Museu Paraense Emílio Göeldi, sér Ciências Humanas 2005 1 149 169

[B2] Rist J Milner-Gulland E Cowlishaw G Rowcliffe M Hunter Reporting of Catch per Unit Effort as a Monitoring Tool in a Bushmeat-Harvesting System Información sobre la Captura por Unidad de Esfuerzo Proporcionada por Cazadores como una Herramienta de Monitoreo en un Sistema de Cosecha de Carne Silvestre Conservation Biology 2010 24 489 499 10.1111/j.1523-1739.2010.01470.x 20491849

[B3] Foster MS James SR Dogs, Deer, or Guanacos: Zoomorphic Figurines from Pueblo Grande, Central Arizona Journal of Field Archaeology 2002 29 165 176 10.2307/3181491

[B4] Frazier J Sustainable use of wildlife: The view from archaeozoology Journal for Nature Conservation 2007 15 163 173 10.1016/j.jnc.2007.08.001

[B5] Alvard MS Robinson JG Redford KH Kaplan H The Sustainability of Subsistence Hunting in the Neotropics Conservation Biology 1997 11 977 982 10.1046/j.1523-1739.1997.96047.x

[B6] Alves RRN Mendonça LET Confessor MVA Vieira WLS Lopez LCS Hunting strategies used in the semi-arid region of northeastern Brazil Journal of Ethnobiology and Ethnomedicine 2009 5 1 50 10.1186/1746-4269-5-1 19386121 PMC2678999

[B7] Ikeya K Hunting with Dogs among the San in the Central Kalahari African Study Monographs 1994 15 119 134

[B8] Larsen CS Animal source foods and human health during evolution The Journal of nutrition 2003 133 3893 3897 10.1093/jn/133.11.3893S 14672287

[B9] Holzman D Meat eating is an old human habit New Scientist 2003 179

[B10] Wing ES Kiple KF, Ornelas KC Animals used for food in the past: As seen by their remains excavated from archeological sites The Cambridge world history of food 2000 1 Cambridge: Cambridge University Press 51 58

[B11] Serpell J In the company of animals: A study of human-animal relationships 1996 Cambridge: Cambridge University Press

[B12] Marques JGW Pescando pescadores: etnoecologia abrangente no baixo São Francisco alagoano 1995 São Paulo, BR: NUPAUB-USP

[B13] Sax B The Mythological Zoo: An Encyclopedia of Animals in World Myth, Legend and Literature 2002 Santa Barbara,: ABC-CLIO, Inc

[B14] Alves RRN Souto WMS Alves RRN, Souto WMS, Mourão JS Etnozoologia: conceitos, considerações históricas e importância A Etnozoologia no Brasil: Importância, Status atual e Perspectivas 2010 7 1 Recife, PE, Brazil: NUPEEA 19 4021461541

[B15] Sillitoe P Ethnobiology and applied anthropology: rapprochement of the academic with the practical Journal of the Royal Anthropological Institute 2006 12 S119 S142 10.1111/j.1467-9655.2006.00276.x

[B16] Von Ihering R Ensaio geográfico sôbre o vocabulário zoológico popular do Brasil Revista Brasileira de Geografia 1939 3 73 88

[B17] Ribeiro R A triste e malsucedida epopéia transatlântica da onça que "morreo de raiveza, ferrando os dentes em hum pao" O tráfico de animais no Brasil Colônia Book A triste e malsucedida epopéia transatlântica da onça que "morreo de raiveza, ferrando os dentes em hum pao" O tráfico de animais no Brasil Colônia 2006

[B18] Papavero N Os 500 anos da Zoologia no Brasil Ciência Hoje 2000 28 30 3521774139

[B19] Nomura H História da Zoologia no Brasil - século XVI 1996 1 Mossoró, RN: FundaçãoVingt-un Rosado e ETFRN-UNED21766508

[B20] Papavero N Teixeira DM Braz da Costa Rubim e seu mini-dicionário de nomes indígenas dos animais do Brasil (1882) Contribuições Avulsas sobre a História Natural do Brasil 2000 32 1 2

[B21] Papavero N Teixeira DM Luz JRP A fauna da Amazônia brasileira nos relatos de viajantes e cronistas dos séculos XVI a XVIII. 2. A viagem de Orellana rio Amazonas abaixo nos anos de 1541 e 1542 e a crônica de Frei Gaspar de Carvajal Contribuições Avulsas sobre a História Natural do Brasil 1999 8 1 6

[B22] Papavero N Teixeira DM A fauna do Maranhão segundo a "Poranduba Maranhense" de Frei Francisco de N. S. dos Prazeres Maranhão" (1820) Contribuições Avulsas sobre a História Natural do Brasil 2000 40 1 14

[B23] Papavero N Teixeira DM Informações zoológicas contidas nas Cartas de Luiz dos Santos Vilhena (Fins do século XVIII) Contribuições Avulsas sobre a História Natural do Brasil 2000 25 1 10

[B24] Papavero N Teixeira DM Overal WL Luz JRP O Novo Éden. A fauna da Amazônia brasileira nos relatos de viajantes e cronistas desde a descoberta do rio Amazonas por Pinzón (1500) até o Tratado de Santo Ildsefonso (1777) 2002 2 Belém, PA, Brazil: Museu Paraense Emílio Gopeldi & MCT21742928

[B25] Nomura H História da Zoologia no Brasil - século XVIII 1998 1 Lisboa, Portugal: Museu Bocage - Museu Nacional de História Natural21766508

[B26] Oliveira FC Albuquerque UP Fonseca-Kruel VS Hanazaki N Avanços nas pesquisas etnobotânicas no Brasil Acta Botanica Brasilica 2009 23

[B27] Salick J Alcorn J Anderson E Asa C Balee W Balick M Beckerman S Bennett B Caballero J Camilo G Intellectual Imperatives in Ethnobiology: NSF Biocomplexity Workshop Report St Louis: Missouri Botanical Gardens 2003 http://www econbot org/pdf/NSF_brochure pdf

[B28] Ribeiro BG Ribeiro D Suma etnológica brasileira 1986 Vozes21701765

[B29] Medeiros MFST Garcia LG O consumo e as estratégias de caça utilizadas pelas populações tradicionais da Reserva Extrativista Chico Mendes Interações 2006 3 121 13421447906

[B30] Fuccio H Carvalho EF Vargas G Perfil da caça e dos caçadores no estado do Acre, Brasil Revista Aportes Andinos 2003 6 1 18

[B31] Pezzuti JCB Silva DF Rebelo GH Lima JP Silva FPC, Gomes-Silva DA, Melo JS, Nascimento VM A captura de quelônios no Parque Nacional do Jaú, Amazonas Coletânea de Textos: manejo e monitoramento de fauna silvestre em florestas tropicais 2008 Belém; 150 15621743943

[B32] Ramos RM Carmo NS Pezzuti JCB Monteiro MA Caça e uso da fauna Atlas socioambiental: municípios de Tomé-Açu, Aurora do Pará, Ipixuna do Pará, Paragominas e Ulianópolis 2008 Belém: NAEA 224 232

[B33] Ayres JM Ayres C Aspectos da caça no alto rio Aripuanã Acta Amazônica 1979 9 287 298

[B34] Smith NJH Utilization of game along Brazil's transamazon highway Acta Amazonica 1976 6 455 466

[B35] Smith NJH Human exploitation of terra firme fauna in Amazonia Ciência e Cultura 1978 30 17 2321774139

[B36] Smith NJH Caimans, Capybaras, otters, manatees, and man in amazonia Biological Conservation 1981 19 177 187 10.1016/0006-3207(81)90033-1

[B37] Ayres JM Lima D Martins ES Barreiros JL Robinson JG, Redford KH On the track of the road: changes in subsistence hunting in a Brazilian Amazonian Village Neotropical wildlife use and conservation 1991 Chicago, USA: University Press 82 92

[B38] Trinca CT Ferrari SF Jacobi P, Ferreira LC Caça em assentamento rural na Amazônia matogrossense Diálogos em ambiente e sociedade no Brasil 2006 1 Indaiatuba, SP: ANPPAS 155 16721774122

[B39] Baia Júnior P Guimarães DAA Le Pendu Y Non-legalized commerce in game meat in the Brazilian amazon: a case study Revista de Biología Tropical 2010 58 1079 1088 10.15517/rbt.v58i2.5264 20737856

[B40] Fachín-Terán A Vogt RC Thorbjarnarson JB Silvius K, Bodmer RE, Fragoso JMV Patterns of Use and Hunting of Turtles in the Mamirauá Sustainable Development Reserve, Amazonas, Brazil People in nature: wildlife conservation in South and Central America 2004 1 New York, USA: Columbia University Press 363 377

[B41] Carvalho EAR Pezzuti JCB Hunting of jaguars and pumas in the Tapajós-Arapiuns Extractive Reserve, Brazilian Amazonia Oryx 2010 44 610 612 10.1017/S003060531000075X

[B42] Albuquerque HN Albuquerque ICS Menezes IR Monteiro JA Barbosa AR Cavalcanti MLF Utilização da Maniçoba (Manihot glaziowii Mull., Euphorbiaceae) na caça de aves em Sertânia-PE Revista de Biologia e Ciências da Terra 2004 4 2 1 6

[B43] Ramos MM Mourão JS Abrantes SHF Conhecimento tradicional dos caçadores de Pedra Lavrada (Paraíba, Brasil) sobre os recursos faunísticos caçados Sitientibus Série Ciências Biológicas 2009 9 215 22421774140

[B44] Barbosa JAA Nobrega VA Alves RRN Aspectos da caça e comércio ilegal da avifauna silvestre por populações tradicionais do semi-árido paraibano Revista de Biologia e Ciências da Terra 2010 2 39 49

[B45] Alves RRN Mendonça LET Confessor MVA Vieira WLdS Vieira KS Alves FN Alves RRN, Souto WMS, Mourão JS Caça no semi-árido paraibano: uma abordagem etnozoológica A Etnozoologia no Brasil: Importância, Status atual e Perspectivas 2010 7 1 Recife, PE, Brazil: NUPEEA 347 37821461541

[B46] Miranda CL Alencar GS Aspects of hunting activity in Serra da Capivara National Park, in the state of Piauí, Brazil Natureza & Conservação 2007 5 115 12121668601

[B47] Dantas-Aguiar PR Barreto RM Santos-Fita D Santos EB Hunting Activities and Wild Fauna Use: a Profile of Queixo D'antas Community, Campo Formoso, Bahia, Brazil Bioremediation, Biodiversity and Bioavailability 2011

[B48] Barboza RRD Mourão JS Souto WMS Alves RRN Knowledge and Strategies of Armadillo (Dasypus novemcinctus L. 1758 and Euphractus sexcinctus L. 1758) Hunters in the "Sertão Paraibano", Paraíba State, NE Brazil Bioremediation, Biodiversity and Bioavailability 2011 5 1 7

[B49] Barbosa JAA Nobrega VA Alves RRN Hunting practices in the semiarid region of Brazil Indian Journal of Traditional Knowledge 2011 10 486 490

[B50] Andriguetto-Filho JM Krüger AC Lange MB Caça, biodiversidade e gestão ambiental na Área de Proteção Ambiental de Guaraqueçaba, Paraná, Brasil Biotemas 1998 11 133 156

[B51] Verdade LM Campos CB How much is a puma worth? Economic compensation as an alternative for the conflict between wildlife conservation and livestock production in Brazil Biota Neotropica 2004 4 1 4

[B52] Pereira JPR Schiavetti A Conhecimentos e usos da fauna cinegética pelos caçadores indígenas "Tupinambá de Olivença" (Bahia) Biota Neotropica 2010 10 175 183 10.1590/S1676-06032010000100018

[B53] Hanazaki N Alves R Begossi A Hunting and use of terrestrial fauna used by Caicaras from the Atlantic Forest coast (Brazil) Journal of Ethnobiology and Ethnomedicine 2009 5 1 36 10.1186/1746-4269-5-1 19930595 PMC2784433

[B54] Lustig-Arecco V Recursos Naturais e Técnicas de Caça Revista de Antropologia 1979 22 39 60

[B55] Cymerys M Shanley P Luz L Quando a caça conserva a mata Ciência Hoje 1997 22 22 2421774139

[B56] Baía Júnior PC Guimarães DAA Parque Ambiental de Belém: um estudo da conservação da fauna silvestre local e a interação desta atividade com a comunidade do entorno Revista Científica da UFPA 2004 4 1 18

[B57] McGrath DG Calabria J Amaral B Futemma CFC Varzeiros, geleiros e o manejo dos recursos naturais na várzea do Baixo Amazonas Cadernos do NAEA 1993 1 91 125

[B58] McGrath DG Castro F Futemma CFC Amaral BD Calabria J Fisheries and the evolution of resource management on the lower Amazon floodplain Human Ecology 1993 21 167 195 10.1007/BF00889358

[B59] Costa-Neto EM Gouw MS Atitudes dos estudantes do Curso de Ciências Biológicas da Universidade Estadual de Feira de Santana (Bahia) com relação à utilização de insetos em atividades didático-científicas Sitientibus Série Ciências Biológicas 2006 6 76 8321774140

[B60] Lima KEC Mayer M Carneiro-Leão AM Vasconcelos SD Conflito ou convergência? percepções de professores e licenciandos sobre ética no uso de animais no ensino de zoologia Investigações em Ensino de Ciências 2008 13 353 36921738046

[B61] Razera JCC Boccardo L Paula J Pereira R Percepções sobre a fauna em estudantes indígenas de uma tribo tupinambá no Brasil: um caso de etnozoologia Revista Electrónica de Enseñanza de las Ciencias 2006 5 466 480

[B62] Pires MRS Pinto LCL Mateus MB Alves RRN, Souto WMS, Mourão JS Etnozoologia como instrumento para a conservação da fauna da Serra do Ouro Branco, Minas Gerais A Etnozoologia no Brasil: Importância, Status atual e Perspectivas 2010 7 1 Recife, PE, Brazil: NUPEEA 471 49421461541

[B63] Begossi A Temporal stability in fishing spots: conservation and co-management in Brazilian artisanal coastal fisheries Ecology and Society 2006 11 5

[B64] Barbosa SRCS Begossi A Fisheries, gender, and local changes at Itaipu Beach, Rio de Janeiro, Brazil: an individual approach Revista Multiciência 2 1 1421755223

[B65] Burda CL Schiavetti A Análise ecológica da pesca artesanal em quatro comunidades pesqueiras da Costa de Itacaré, Bahia, Brasil: Subsídios para a Gestão Territorial Revista da Gestão Costeira Integrada 2008 8 149 168

[B66] Costa-Neto EM Sustainable development and traditional knowledge: a case study in a Brazilian artisanal fishermen's community Sustainable Development 2000 8 89 95 10.1002/(SICI)1099-1719(200005)8:2<89::AID-SD130>3.0.CO;2-S

[B67] Costa-Neto EM Cultura pesqueira, desenvolvimento e sustentabilidade no litoral norte do estado da Bahia: um estudo de caso TecBahia 1999 14 131 139

[B68] Jankowsky M Pires JSR Nordi N Contribuição ao manejo participativo do Caranguejo-uçá, *Ucides cordatus *(L., 1763) Cananéia, SP Boletim do Instituto de Pesca 2006 32 221 22821768542

[B69] Lopes PFM Begossi A Temporal changes in caiçara artisanal fishing and alternatives for management: a case study on the southeastern Brazilian coast Biota Neotropica 2008 8 53 62

[B70] Bruno M Kraemer BM Percepções de estudantes da 6^a ^série (7° ano) do "Ensino Fundamental" em uma escola pública de Belo Horizonte, MG sobre os morcegos: uma abordagem etnozoológica Revista Científica do Departamento de Ciências Biológicas, Ambientais e da Saúde 2010 2 42 50

[B71] Ferreira AM Soares CAAA Aracnídeos peçonhentos: análise das informações nos livros didáticos de ciências Ciência & Educação 2008 14 307 31421774139

[B72] Pezzuti JCB Manejo de caça e a conservação da fauna silvestre com participação comunitária Papers do NAEA (UFPA) 2009 1

[B73] Romão JA Boccardo L Souza M Abordagem dos miriápodos em livros didáticos de ciências Sitientibus Série Ciências Biológicas 2008 8 89 9821774140

[B74] Machado D Catadoras de caranguejo e saberes tradicionais na conservação de manguezais da Amazônia brasileira Estudos Feministas 2007 15 485 490 10.1590/S0104-026X2007000200016

[B75] Costa-Neto EM O caranguejo-de-água-doce, Trichodactylus fluviatilis (Latreille, 1828) (Crustacea, Decapoda, Trichodactylidae), na concepção dos moradores do povoado de Pedra Branca, Bahia, Brasil Biotemas 2007 20 59 68

[B76] Montenegro SCS Marques JGW Nordi N Oliveira FB Pescadores de Camarão no Baixo São Francisco: Abordagem Etnoecológica com Ênfase nas Estratégias de Pesca Conhecimento Tradicional e Estratégias de Sobrevivência de Populações Brasileiras 2007 Macéio, AL, Brazil: EDUFAL 1 157

[B77] Montenegro SCS Nordi N Marques JGW Contexto cultural, ecológico e econômico da produção e ocupação dos espaços de pesca pelos pescadores de pitu (Macrobrachium carcinus) em um trecho do Baixo São Francisco, Alagoas-Brasil Interciencia 2001 26 535 540

[B78] Magalhães HF Costa Neto EM Schiavetti A Saberes pesqueiros tradicionais relacionados à coleta de crustáceos (Decapoda: Brachyura) no município de Conde, estado da Bahia Biota Neopropica 2011 11 1 10

[B79] Alves RRN Nishida AK A ecdise do caranguejo-uçá, Ucides cordatus L. (DECAPODA, BRACHYURA) na visão dos caranguejeiros Interciencia 2002 27 110 117

[B80] Costa-Neto EM Lima KLG Contribuição ao estudo da interação entre pescadores e caranguejos (Crustacea, Decapoda, Brachyura): considerações etnobiológicas em uma comunidade pesqueira do estado da Bahia, Brasil Actualidades Biologicas 2000 22 195 202

[B81] Fiscarelli AG Pinheiro MAA Perfil sócio-econômico e conhecimento etnobiológico do catador do caranguejo-uçá, *Ucides cordatus *(Linnaeus, 1763) nos manguezais de Iguape (24 41S), SP, Brasil Actualidades Biologicas 2002 24 129 142

[B82] Nordi N A captura do caranguejo-uçá (*Ucides cordatus*) durante o evento reprodutivo da espécie: o ponto de vista dos caranguejeiros Revista Nordestina de Biologia 1994 9 41 47

[B83] Nordi N A produção dos catadores do caranguejo-uçá (*Ucides cordatus*) da região de Várzea Nova, Paraíba Revista Nordestina de Biologia 1994 9 71 77

[B84] Nordi N O processo de comercialização do caranguejo-uçá (*Ucides cordatus*) e seus reflexos nas atividades de coleta Revista Nordestina de Biologia 1995 10 39 45

[B85] Nordi N Time allocation and energy expenditure related to crab gathering activity Ciência e Cultura 1997 49 136 13921774139

[B86] Nordi N Nishida AK Alves RRN Effectiveness of Two Gathering Techniques for Ucides cordatus in Northeast Brazil: Implications for the Sustainability of Mangrove Ecosystems Human Ecology 2009 37 121 127 10.1007/s10745-009-9214-9

[B87] Souto FJB Uma abordagem etnoecológica da pesca do caranguejo, *Ucides cordatus*, Linnaeus, 1763 (Decapoda: Brachyura), no manguezal do Distrito de Acupe (Santo Amaro-BA) Biotemas 2007 20 69 80

[B88] Souto FJB Marques JGW "O siri labuta muito!" Uma abordagem etnoecológica abrangente da pesca de um conjunto de crustáceos no manguezal de Acupe, Santo Amaro, Bahia, Brasil Sitientibus Série Ciências Biológicas 2006 6 106 11921774140

[B89] Passos CA Di Beneditto APM Captura comercial do caranguejo-uçá, *Ucides cordatus *(L., 1763), no Manguezal de Gargaú, RJ Biotemas 2005 18 223 231

[B90] Mendonça JT Pereira ALC Avaliação das capturas de caranguejo-uçá *Ucides cordatus *no município de Iguape, litoral sul de São Paulo, Brasil Boletim do Instituto de Pesca 2009 35 169 179

[B91] Carvalho HRL Igarashi MA A utilização do forjo na captura do caranguejo uçá (*Ucides cordatus*) na comunidade de Tapebas em Fortaleza - CE Biotemas 2009 22 69 74

[B92] Silva-Cavalcanti JS Costa MF Fisheries in Protected and Non-Protected Areas: Is it Different? The Case of Anomalocardia Brasiliana at Tropical Estuaries of Northeast Brazil Journal of Coastal Research 2009 1454 1458

[B93] Severino-Rodrigues E Pita JB Graça-Lopes R Pesca artesanal de siris (Crustacea, Decapoda, Portunidae) na região estuarina de Santos e São Vicente (SP), Brasil Boletim do Instituto de Pesca, São Paulo 2001 27 7 19

[B94] Souto FJB Alves RRN, Souto WMS, Mourão JS Etnozoologia na pesca de camarões no manguezal de Acupe, Santo Amaro, Bahia A Etnozoologia no Brasil: Importância, Status atual e Perspectivas 2010 7 1 Recife, PE, Brazil: NUPEEA 193 21021461541

[B95] Oliveira LEC Begossi A Last Trip Return Rate Influence Patch Choice Decisions of Small-Scale Shrimp Trawlers: Optimal Foraging in São Francisco, Coastal Brazil Human Ecology 2011 39 323 332 10.1007/s10745-011-9397-8

[B96] Nomura H Os crustáceos na cultura popular 2001 Mossoró, RN: Fundação Guimarães Duque e Fundação Vingt-un Rosado19995091

[B97] Camargo JMF Posey DA O conhecimento dos Kayapó sobre as abelhas sociais sem ferrão (Meliponidae, Apidae, Hymenoptera): notas adicionais Boletim do Museu Paraense Emílio Goeldi Nova série Zoologia 1990 6 17 42

[B98] Coimbra CEA Jr Estudos de Ecologia Humana entre os Suruí do parque indígena Aripuanã, Rondônia: 1. O uso de larvas de Coleópteros (Bruchidae e Curculionidae) na alimentação Revista Brasileira de Zoologia 1983 2 35 47

[B99] Modro AFH Costa MS Maia E Aburaya FH Percepção entomológica por docentes e discentes do município de Santa Cruz do Xingu, Mato Grosso, Brasil Biotemas 2009 22 153 159

[B100] Posey DA Kayapó controla inseto com uso adequado do ambiente Revista Atualidade Indígena 1979 3 47 56

[B101] Posey DA Wasps, warriors, and fearless men: ethnoentomology of the Kayapó Indians of central Brazil Journal of Ethnobiology 1981 1 165 174

[B102] Posey DA A apicultura popular dos Kayapó Revista Atualidade Indígena 1981 20 36 41

[B103] Posey DA The Importance of Bees to Kayapo Indians of the Brazilian Amazon The Florida Entomologist 1982 65 452 458 10.2307/3494679

[B104] Posey DA Ethnomethodology as an emic guide to cultural systems: the case of the insects and the Kayapó Indians of Amazonia Revista Brasileira de Zoologia 1983 1 135 144

[B105] Posey DA Folk Apiculture of the Kayapo Indians of Brazil Biotropica 1983 15 154 158 10.2307/2387963

[B106] Posey DA Ribeiro D Etnoentomologia de tribos indígenas da Amazônia Suma Etnológica Brasileira Etnobiologia 1986 Petrópolis, RJ: Vozes/Finep 251 27221701765

[B107] Santos-Fita D Costa-Neto EM Schiavetti A Constitution of ethnozoological semantic domains: meaning and inclusiveness of the lexeme" insect" for the inhabitants of the county of Pedra Branca, Bahia State, Brazil Anais da Academia Brasileira de Ciências 2011 83 589 598 10.1590/s0001-37652011000200018 21670881

[B108] Costa-Neto EM As cigarras (Hemiptera: Cicadidae) na visao dos moradores do povoado de Pedra Branca, Bahia, Brasil Boletín de la SEA 2008 43 453 45721216839

[B109] Costa-Neto EM Biotransformações de insetos no povoado de Pedra Branca, Estado da Bahia, Brasil Interciencia 2004 29 280 283

[B110] Costa-Neto EM Bird-spiders (Arachnida, Mygalomorphae) as perceived by the inhabitants of the village of Pedra Branca, Bahia State, Brazil Journal of Ethnobiology and Ethnomedicine 2006 2 7 10.1186/1746-4269-2-7 17101055 PMC1654147

[B111] Costa-Neto EM Centopéias (Arthropoda, Chilopoda) na concepção dos moradores do povoado de Pedra Branca, Bahia, Brasil Boletín de la SEA 2006 39 441 44521216839

[B112] Costa-Neto EM Cricket singing means rain: semiotic meaning of insects in the district of Pedra Branca, Bahia State, northeastern Brazil Anais da Academia Brasileira de Ciências 2006 78 59 68 10.1590/s0001-37652006000100007 16532207

[B113] Costa-Neto EM *Fulgora laternaria *Linnaeus, 1758 (Hemiptera: Fulgoridae) na concepção dos moradores do povoado de Pedra Branca, Santa Terezinha, Bahia, Brasil Revista de Ciências Ambientais 2007 1 36 5620863933

[B114] Costa-Neto EM Insetos como recursos alimentares nativos no semi-árido do estado da Bahia, nordeste do Brasil Zonas Áridas 2004 8 33 4021764137

[B115] Costa-Neto EM La etnoentomología de las avispas (Hymenoptera, Vespoidea) en el poblado de Pedra Branca, estado de Bahia, nordeste de Brasil Boletín de la SEA 2004 247 26221216839

[B116] Costa-Neto EM O conhecimento etnoentomológico do cavalo-do-cão (Hymenoptera, Pompilidae) no povoado de Pedra Branca, estado da Bahia, Brasil Rev bra Zoociências 2004 6 249 26016460148

[B117] Costa-Neto EM Os insetos que ofendem: artropodoses na visão dos moradores da região da Serra da Jibóia, Bahia, Brasil Sitientibus Série Ciências Biológicas 2004 4 81 9021774140

[B118] Costa-Neto EM "Piolho-de-cobra" (Arthropoda: Chilopoda: Geophilomorpha) na concepção dos moradores de Pedra Branca, Santa Terezinha, Estado da Bahia, Brasil Acta Scientiarum Biological Sciences 2008 28 143 148

[B119] Costa-Neto EM The perception of Diplopoda (Arthropoda, Myriapoda) by the inhabitants of the county of Pedra Branca, Santa Teresinha, Bahia, Brasil Acta Biologica Colombiana 2007 12 125 136

[B120] Costa-Neto EM Carvalho PD Percepção dos insetos pelos graduandos da Universidade Estadual de Feira de Santana, Bahia, Brasil Acta Scientiarum Biological Sciences 2000 22 423 428

[B121] Costa-Neto EM Lago APA Martins CC Júnior PB O "Louva-a-Deus-de-cobra", *Phibalosoma sp*. (Insecta, Phasmida), segundo a percepção dos moradores de Pedra Branca, Santa Terezinha, Bahia, Brasil Sitientibus 2005 5 1 33 38

[B122] Costa-Neto EM Pacheco JM "Head of snake, wings of butterfly, and body of cicada": impressions of the lantern-fly (Hemiptera: Fulgoridae) in the village of Pedra Branca, Bahia State, Brazil Journal of Ethnobiology 2003 23 23 46

[B123] Costa-Neto EM Pacheco JM A construção do domínio etnozoológico "inseto" pelos moradores do povoado de Pedra Branca, Santa Terezinha, Estado da Bahia Acta Scientiarum Biological Sciences 2004 26 81 90

[B124] Costa-Neto EM Resende JJ A percepção de animais como "insetos" e sua utilização como recursos medicinais na cidade de Feira de Santana, Estado da Bahia, Brasil Acta Scientiarum 2004 26 143 149

[B125] Costa-Neto EM Rodrigues RMFR As formigas (Insecta: Hymenoptera) na concepção dos moradores de Pedra Branca, Santa Terezinha, estado da Bahia, Brasil Boletín de la SEA 2005 353 36421216839

[B126] Costa-Neto EM Rodrigues RMFR Os besouros (Insecta: Coleoptera) na concepção dos moradores de Pedra Branca, Santa Terezinha, Estado da Bahia Acta Scientiarum Biological Sciences 2006 28 1 71 80

[B127] Costa-Neto EM Sánchez-Salinas S Actitudes de los estudiantes de licenciatura en lengua castellana de la Universidad Estatal de Feria de Santana, Bahia, Brasil, en relación con los insectos comestibles Diálogos & Ciência 2008 16 39 4721774122

[B128] Dias MA Costa-Neto EM "Grilos" (Orthoptera) na percepção dos moradores de Feira de Santana, Bahia Sitientibus Série Ciências Biológicas 2005 5 99 11421774140

[B129] Sampaio JA Castro MS Silva FO Uso da cera de abelhas pelos índios Pankararé no Raso da Catarina, Bahia, Brasil Arquivos do Museu Nacional 2009 67 3 12

[B130] Costa-Neto EM Andrade JN Fuentes AM, Silva MTP, Méndez RM, Azúa RV, Correa PM, Santillán TVG Dimensão cognitiva, afetiva e comportamental da interação dos seres humanos com as lagartas (Insecta: Lepidoptera) no município de Feira de Santana, Bahia, Brasil Sistemas biocognitivos tradicionales: Paradigmas en la conservación biológica y el fortalecimiento cultural 2010 1 Pachuca, Mexico: Universidad Autónoma del Estado de Hidalgo, Asociación Etnobiológica Mexicana y Sociedad Latinoamericana de Etnobiología 3 8

[B131] Santos LO Gurgel-Gonçalves R Damasceno CP Costa-Neto EM Os piolhos-da-cabeça (Phthiraptera: Pediculidae) na visão de mães e filhos usuários de postos de assistência no Distrito Federal, Brasil Boletín de la SEA 2009 45 575 57821216839

[B132] Modro AFH Souza S Aburaya FH Maia E Conhecimento dos moradores do médio Araguaia, Estado do Mato Grosso, sobre a utilidade de produtos de abelhas (Hymenoptera, Apidae) Acta Scientiarum Biological Sciences 2009 31 421 424

[B133] Ulyssea MA Hanazaki N Lopes BC Insetos no folclore da comunidade do Ribeirão da Ilha, Florianópolis Sitientibus 2010 10 244 251

[B134] Costa-Neto E Magalhães HF The ethnocategory ''insect'' in the conception of the inhabitants of Tapera County, São Gonçalo dos Campos, Bahia, Brazil Anais da Academia Brasileira de Ciências 2007 79 239 249 10.1590/S0001-37652007000200007 17625679

[B135] Rodrigues AS Até quando o etnoconhecimento sobre as abelhas sem ferrão (Hymenoptera, Apidae, Meliponinae) será transmitido entre gerações pelos índios Guarani M'byá da Aldeia Morro da Saudade, localizada na cidade de São Paulo, Estado de São Paulo, Brasil? Sitientibus Série Ciências Biológicas 2006 6 343 35021774140

[B136] Boccardo L Costa-Neto EM Silva TRd Jucá-Chagas R Costa-Neto EM, Alves RRN Insetos na medicina popular do povoado de Porto Alegre, Maracás, Bahia Zooterapia: Os Animais na Medicina Popular Brasileira 2010 2 1 Recife, PE, Brazil: NUPEEA 209 220

[B137] Carrera M Nota sobre insetos utilizados como adorno Revista Brasileira de Entomologia 1982 26 133 135

[B138] Carrera M Insetos, lendas e história 1991 Thesaurus

[B139] Carrera M Entomofagia humana Revista Brasileira de Entomologia 1992 36 889 894

[B140] Coimbra CEA Jr Santos RV Bicudo das palmáceas: praga ou alimento Ciência Hoje, Rio de Janeiro 1993 16 59 6021774139

[B141] Costa-Neto EM Motte-Florac É, Thomas JMC Considerations on the man/insect relationship in the state of Bahia, Brazil Les "insectes" dans la tradition orale 2003 Paris-Louvain: Peeters-SELAF 95 10421771436

[B142] Costa-Neto EM Estudos etnoentomológicos no estado da Bahia, Brasil: uma homenagem aos 50 anos do campo de pesquisa Biotemas 2004 17 117 149

[B143] Costa-Neto EM Insetos como fontes de alimentos para o homem: Valoração de recursos considerados repugnantes Interciencia 2003 28 136 140

[B144] Costa-Neto EM Ramos-Elorduy J Los Insectos Comestibles de Brasil: Etnicidad, Diversidad e Importancia en la Alimentación Boletín Sociedad Entomológica Aragonesa 2006 423 44221216839

[B145] Costa-Neto EM Ramos-Elorduy J Pino JM Los insectos medicinales de Brasil: Primeros resultados Boletín Sociedad Entomológica Aragonesa 2006 395 41421216839

[B146] Costa-Neto EM O significado dos Orthoptera (Arthropoda, Insecta) no Estado de Alagoas Sitientibus 1998 18 9 17

[B147] Costa Neto EM A etnocategoria "inseto" e a hipótese da ambivalência entomoprojetiva Acta Biológica Leopoldensia 1999 2 7 1421660722

[B148] Lenko K Papavero N Insetos no Folclore 1996 2 São Paulo, Brazil: Plêiade/FAPESP

[B149] Lenko K Papavero N Os insetos no folclore 1979 1 São Paulo, Brazil: Conselho Estadual de Artes e Ciências Humanas

[B150] Posey DA Topics and issues in ethnoentomology with some suggestions for the development of hypothesis-generation and testing in ethnobiology Journal of Ethnobiology 1986 6 99 120

[B151] Matthiensen FA Os escorpiões e suas relações com o homem: uma revisão Ciência e Cultura 1988 40 1168 117221774139

[B152] Pacheco JM Etnoentomologia: o que é um inseto? Informativo da Sociedade Entomológica do Brasil 2001 26 1 5

[B153] Posey DA Temas e inquirições em etnoentomologia: algumas sugestões quanto à geração de hipóteses Boletim do Museu Paraense Emílio Goeldi 1987 3 99 134

[B154] Posey DA Ethnoentomological survey of Brazilian Indians Entomology General 1987 12 191 202

[B155] Nomura H Os animais no folclore - aracnídeos e miriápodos 2001 Mossoró, RN: Fundação Guimarães Duque e Fundação Vingt-un Rosado

[B156] Nomura H Curiosidades folclóricas sobre insetos 2001 São José dos Campos, SP: Centro de Estudos da Cultura Popular

[B157] Almeida DF Barros CS Etnomiriapodologia: Os embuás sob o ponto de vista cultural em contexto educativo Revista Eletrônica de Humanidades do Curso de Ciências Sociais da UNIFAP 2009 2 without page numbers

[B158] Silva T Boccardo L Costa-Neto EM Jucá Chagas R Os saberes dos moradores do povoado de Porto Alegre (Maracás, Bahia, Brasil) sobre os insetos Boletín de la SEA 2010 46 603 60821216839

[B159] Ulysséa MA Hanazaki N Lopes BC Percepção e uso dos insetos pelos moradores da comunidade do Ribeirão da Ilha, Santa Catarina, Brasil Revista Biotemas 2010 23 3 191 202

[B160] Pezzuti JCB Lima JP Silva DF Begossi A Uses and Taboos of Turtles and Tortoises Along Rio Negro, Amazon Basin Journal of Ethnobiology 2010 30 153 168 10.2993/0278-0771-30.1.153

[B161] Pezzuti JCB Barboza RSL Nunes I Miorando P Fernandes L Alves RRN, Souto WMS, Mourão JS Etnoecologia e conservação de quelônios amazônicos: um estudo de caso A Etnozoologia no Brasil: Importância, Status atual e Perspectivas 2010 7 1 Recife, PE, Brazil: NUPEEA 447 47021461541

[B162] Alves ÂGC Souto FJB Leite AM Etnoecologia dos cágados-d'água Phrynops spp. (Testudinomorpha: Chelidae) entre pescadores artesanais do açúde de Bodocongó, Campina Grande, Paraíba, Nordeste do Brasil Sitientibus 2002 2 62 68

[B163] Barbosa AR Nishida AK Costa ES Cazé ALR Abordagem etnoherpetológica de São José da Mata - Paraíba - Brasil Revista de Biologia e Ciências da Terra 2007 7 117 123

[B164] Santos-Fita D Costa-Neto EM Schiavetti A 'Offensive' snakes: cultural beliefs and practices related to snakebites in a Brazilian rural settlement Journal of Ethnobiology and Ethnomedicine 2010 6 1 13 10.1186/1746-4269-6-1 20346120 PMC2853519

[B165] Magalhães L A cobra eo folclore sertanejo Revista do Instituto do Ceará 1969 87 113 123

[B166] Marques JGW Guerreiro W Répteis em uma Feira Nordestina (Feira de Santana, Bahia). Contextualização Progressiva e Análise Conexivo-Tipológica Sitientibus Série Ciências Biológicas 2007 7 289 29521774140

[B167] Costa-Neto EM Valverde MCC Baptista GCS Diálogo entre concepções prévias dos estudantes e conhecimento científico escolar: relações sobre os Amphisbaenias Revista Iberoamericana de Educación (Online) 2008 4718473570

[B168] Mendes EG Sapos: ficção e ciência Ciência e Cultura 1987 39 56 6021774139

[B169] Nomura H Usos e crendices sobre anfíbios 1996 1 Mossoró, RN, Brazil: Fundação Vingt-un Rosado

[B170] Nomura H Os répteis no folclore 1996 1 Mossoró, RN, Brasil: Fundação Vingt-un Rosado21719134

[B171] Alves RRN Pereira-Filho GA Vieira KS Santana GG Vieira WLS Almeida WO Alves RRN, Souto WMS, Mourão JS Répteis e as populações humanas no Brasil: uma abordagem etnoherpetológica A Etnozoologia no Brasil: Importância, Status atual e Perspectivas 2010 7 1 Recife, PE, Brazil: NUPEEA 121 14821461541

[B172] Amaral BD Fishing Territoriality and Diversity Between the Ethnic Populations Ashaninka and Kaxinawá, Breu River, Brazil/Peru Acta Amazonica 2004 34 75 88 10.1590/S0044-59672004000100011

[B173] Batistella AM Castro CP Vale JD Conhecimento dos moradores da comunidade de Boas Novas, no Lago Janauacá - Amazonas, sobre os hábitos alimentares dos peixes da região Acta Amazonica 2005 35 51 54 10.1590/S0044-59672005000100008

[B174] Begossi A Garavello JC Notes on the ethnoichthyology of fishermen from the Tocantins River (Brazil) Acta Amazonica 1990 20 341 351

[B175] Begossi A Silvano RAM Amaral BD Oyakama OT Uses of Fish and Game by Inhabitants of an Extrative Reserve (Upper Juruá, Acre, Brazil) Environment, Development and Sustainability 1999 1 73 93 10.1023/A:1010075315060

[B176] Petrere Júnior M Nota sobre a pesca dos índios Kayapó da aldeia de Gorotire, Rio Fresco, Pará Boletim do Museu Paraense Emílio Goeldi 1990 6 5 17

[B177] Ribeiro BG Kenhíri T Pavan C Etnoictiologia desâna Uma estratégia latino-americana para a Amazônia 1996 1 Brasília, Brazil: Ministério do Meio Ambiente, dos Recursos Hídricos e da Amazônia Legal 201 217

[B178] Silva AL Begossi A Begossi A, Leme A, Seixas CS, Castro F, Pezzuti J, Hanazaki N, Peroni N, Silvano RAM Uso de recursos por ribeirinhos no médio rio Negro Ecologia de Pescadores da Mata Atlântica e da Amazônia 2004 São Paulo, SP, Brazil: Hucitec 185 220

[B179] Brandão FC Silva LMA Conhecimento Ecológico Tradicional dos pescadores da Floresta Nacional do Amapá Uakari 2009 4 55 66

[B180] Costa-Neto EM Dias CV Melo MN O conhecimento ictiológico tradicional dos pescadores da cidade de Barra, região do Médio São Francisco, Estado da Bahia, Brasil Acta Scientiarum 2002 24 561 572

[B181] Moura FBP Marques JGW Conhecimento de pescadores tradicionais sobre a dinâmica espaço-temporal de recursos naturais na Chapada Diamantina, Bahia Biota Neotropica 2007 7 119 126 10.1590/S1676-06032007000300014

[B182] Moura FBP Marques JGW Funch LS, Funch R, Queiroz LP O Povo dos Marimbus: Etnoecologia de pescadores tradicionais na APA Marimbus-Iraquara Serra do Sincora - Parque Nacional da Chapada Diamantina 2008 1 Feira de Santana, BA, Brazil: Radami 213 221

[B183] Moura FBP Marques JGW Nogueira EMS "Peixe sabido, que enxerga de longe": Conhecimento ictiológico tradicional na Chapada Diamantina, Bahia Revista Biotemas 2008 21 115 123

[B184] Begossi A Braga FMS Food taboos and folk medicine among fishermen from the Tocantins River Amazoniana 1992 12 101 118

[B185] Silva TFP Costa-Neto EM Percepção de insetos por moradores da comunidade Olhos D'água, município de Cabaceiras do Paraguaçu, Bahia, Brasil Boln SEA 2004 262 268

[B186] Silvano RAM Begossi A Ethnoichthyology and fish conservation in the Piracicaba River (Brazil) Journal of Ethnobiology 2002 22 285 306

[B187] Silvano RAM Begossi A Seasonal dynamics of fishery at the Piracicaba River (Brazil) Fisheries Research 2001 51 69 86 10.1016/S0165-7836(00)00229-0

[B188] Azevedo-Santos VM Costa-Neto EM Lima-Stripari N Concepção dos pescadores artesanais que utilizam o reservatório de Furnas, Estado de Minas Gerais, acerca dos recursos pesqueiros: um estudo etnoictiológico Revista Biotemas 2010 23 135 145

[B189] Paz VA Begossi A Ethnoichthyology of Galviboa fishermen of Sepetiba Bay, Brazil Journal of Ethnobiology 1996 16 157 168

[B190] Begossi A Figueiredo JL Ethnoichthyology of southern coastal fishermen: cases from Búzios Island and Sepetiba Bay (Brazil) Bulletin of Marine Science 1995 56 710 717

[B191] Begossi A Richerson PJ Biodiversity, family income and ecological niche: a study on the consumption of animal foods on Búzios Island (Brazil) Ecology of Food and Nutrition 1993 30 51 51 10.1080/03670244.1993.9991322

[B192] Begossi A Fishing spots and sea tenure: Incipient forms of local management in Atlantic forest coastal communities Human Ecology 1995 23 387 406 10.1007/BF01190138

[B193] Begossi A The use of optimal foraging theory in the understanding of fishing strategies: A case from Sepetiba Bay (Rio de Janeiro State, Brazil) Human Ecology 1992 20 463 475 10.1007/BF00890430

[B194] Clauzet M Ramires M Begossi A A Etnoictiologia dos pescadores artesanais da Praia de Guaibim, Valença (BA), Brasil Neotropical Biology And Conservation 2007 2 136 154

[B195] Cordell J The lunar-tide fishing cycle in Northeastern Brazil Ethnology 1974 13 379 392 10.2307/3773053

[B196] Costa-Neto EM A cultura pesqueira do Litoral Norte da Bahia: etnoictiologia, desenvolvimento e sustentabilidade 2001 1 Macéio, Brazil: EDUFBA/EDUFAL

[B197] Costa-Neto EM Marques JGW Conhecimento ictiológico tradicional e a distribuição temporal e espacial de recursos pesqueiros pelos pescadores de Conde, Estado da Bahia, Brasil Etnoecológica 2000 4 56 68

[B198] Costa-Neto EM Marques JGW Etnoictiologia dos pescadores artesanais de Siribinha, município de Conde (Bahia): aspectos relacionados com a etologia dos peixes Acta Scientiarum Biological Sciences 2008 22 553 560

[B199] Fernandes-Pinto E Marques JGW Diegues AC Conhecimento Etnoecológico de Pescadores Artesanais de Guaraqueçaba - PR Enciclopédia Caiçara - O Olhar do Pesquisador 2004 1 São Paulo, Brazil: Editora Hucitec 163 190

[B200] Hanazaki N Begossi A Fishing and Niche Dimension for Food Consumption of Caiçaras from Ponta do Almada (Brazil) Human Ecology Review 2000 7 52 62

[B201] Hanazaki N Begossi A Catfish as Mullets: The Food preferences and taboos os caiçaras (Southern Atlantic Forest Coast Interciencia 2006 31 123 129

[B202] Mourão JS Nordi N Etnoictiologia de Pescadores Artesanais do Estuário do Rio Mamanguape, Paraíba, Brasil Boletim do Instituto de Pesca 2003 29 9 17

[B203] Mourão JS Nordi N Pescadores, peixes, espaço e tempo: uma abordagem Etnoecológica Interciencia 2006 31 358 363

[B204] Nehrer R Begossi A Fishing at Copacabana (Rio de Janeiro): local strategies in a global city Ciência e Cultura 2000 52 26 3021774139

[B205] Pacheco RS Marques JGW Restrições à inserção de peixes em cadeias trófico-culturais de uma população pesqueira no Recôncavo Baiano (Acupe, Santo Amaro) Revista Ouricuri 2009 1 91 114

[B206] Ramires M Barrella W Conhecimento popular sobre peixes em uma comunidade caiçara da Estação Ecológica de Juréia Itatins Boletim do Instituto de Pescaogia 2001 27 97 104

[B207] Ramires M Barrella W Ecologia da pesca artesanal em populações caiçaras da Estação Ecológica de Juréia-Itatins, São Paulo, Brasil Interciencia 2003 28 208 213

[B208] Ramires M Barrella W Diegues AC Etnoictiologia de Pescadores da Estação Ecológica de Juréia Itatins Enciclopédia Caiçara 2004 1 1 São Paulo, Brazil: NUPAUB-CEC/HUCITEC

[B209] Ramires M Molina SMG Hanazaki N Etnoecologia caiçara: o conhecimento dos pescadores artesanais sobre aspectos ecológicos da pesca Biotemas 2007 20 101 113

[B210] Rocha MSP Mourão JS Souto WMS Barboza RRD Alves RRN Uso dos recursos pesqueiros no Estuário do Rio Mamanguape, Estado da Paraíba, Brasil Interciencia 2008 33 903 909

[B211] Rosa IL Alves RRN Albuquerque UP, Alves AGC, Araújo TAS Pesca e comércio de cavalos-marinhos (Syngnathidae Hippocampus) no Norte e Nordeste do Brasil: subsídios para conservação e manejo Povos e Paisagens Etnobiologia, Etnoecologia e Biodiversidade no Brasil 2007 1 Recife, PE, Brazil: NUPEEA/UFRPE 28 46

[B212] Rosa I Alves RRN Bonifacio K Mourão JS Osorio F Oliveira T Nottingham M Fishers' knowledge and seahorse conservation in Brazil Journal of Ethnobiology and Ethnomedicine 2005 1 1 12 10.1186/1746-4269-1-1 PMC1334218 16336660

[B213] Seixas CS Begossi A Ethnozoology of fishing communities from Ilha Grande (Atlantic forest coast, Brazil) Journal of Ethnobiology 2001 21 107 135

[B214] Seixas C Begoss A Central Place optimal foraging theory: populations and individual analyses of fishing strategies at Aventureiro (Ilha Grande, Brazil) Ciência e Cultura 2000 52 85 9221774139

[B215] Silvano RAM Begossi A Local knowledge on a cosmopolitan fish Ethnoecology of *Pomatomus saltatrix *(Pomatomidae) in Brazil and Australia Fisheries Research 2005 71 43 59 10.1016/j.fishres.2004.07.007

[B216] Silvano RAM MacCord PFL Lima RV Begossi A When does this fish spawn? Fishermen's local knowledge of migration and reproduction of Brazilian coastal fishes Environmental Biology of Fishes 2006 76 371 386 10.1007/s10641-006-9043-2

[B217] Silvano RAM Begossi A What can be learned from fishers? An integrated survey of fishers' local ecological knowledge and bluefish *(Pomatomus saltatrix*) biology on the Brazilian coast Hydrobiologia 2010 637 3 18 10.1007/s10750-009-9979-2

[B218] Souto FJB O bosque de mangues e a pesca artesanal no Distrito de Acupe (Santo Amaro, Bahia): uma abordagem etnoecológica Acta Scientiarum Biological Sciences 2008 30 275 282

[B219] Reuss-Strenzel GM Assunção MF Etnoconhecimento ecológico dos caçadores submarinos de Ilhéus, Bahia, como subsídio à preservação do mero Revista da Gestão Costeira Integrada 2008 8 203 219

[B220] Begossi A Mapping spots: fishing areas or territories among islanders of the Atlantic Forest (Brazil) Regional Environmental Change 2001 2 1 12 10.1007/s101130100022

[B221] Rosa IML Alves RRN Bonifácio KM Osório F Mourão JS Oliveira TPR Nottingham MC Alves RRN, Souto WMS, Mourão JS Bioecologia de cavalos-marinhos (Teleostei: Syngnathidae Hippocampus) na visão de pescadores do Norte e Nordeste do Brasil A Etnozoologia no Brasil: Importância, Status atual e Perspectivas 2010 7 1 Recife, PE, Brazil: NUPEEA 297 32221461541

[B222] Begossi A Svetlana SV Andreoli TB Clauzet M Martinelli CM Ferreira AGL Oliveira LEC Silvano R Ethnobiology of snappers (Lutjanidae): target species and suggestions for management Journal of Ethnobiology and Ethnomedicine 2011 7 10.1186/1746-4269-7-11 PMC3068939 21410969

[B223] Thé APG Madi E Nordi N Godinho HP, Godinho AL Conhecimento local, regras informais e uso do peixe na pesca do alto-médio São Francisco Águas, peixes e pescadores do São Francisco das Gerais 2003 Belo Horizonte, MG, Brazil: PUCMinas 1 468

[B224] Hanazaki N Begossi A Does Fish Still Matter? Changes In The Diet Of Two Brazilian Fishing Communities Ecology of Food and Nutrition 2003 42 279 301 10.1080/03670240390229643 22260174

[B225] Rossato JC A saúva no folclore paulista Anuário do Folclore 1984 14 1 821733613

[B226] Sampaio FAC Jucá-Chagas R Teixeira PMM Boccardo L Os peixes e a pesca. Concepções de estudantes do povoado de Porto Alegre, Bahia, BA Sitientibus Série Ciências Biológicas 2006 6 44 5721774140

[B227] Silvano RAM Valbo-Jørgensen J Beyond fishermen's tales: contributions of fishers' local ecological knowledge to fish ecology and fisheries management Environment, Development and Sustainability 2008 10 657 675 10.1007/s10668-008-9149-0

[B228] Van Velthem LH Os Wayana, as águas, os peixes e a pesca Boletim do Museu Paraense Emílio Goeldi 1990 6 107 116

[B229] Pinheiro L Da ictiologia ao etnoconhecimento: saberes populares, percepção ambiental e senso de conservação em comunidade ribeirinha do rio Piraí, Joinville, Estado de Santa Catarina Revista Biological Sciences, Maringá 2004 3 325 334

[B230] Lopes P Silvano R Begossi A Extractive and Sustainable Development Reserves in Brazil: resilient alternatives to fisheries? Journal of Environmental Planning and Management 2011 54 421 443 10.1080/09640568.2010.508687

[B231] Rosa IL Oliveira TPR Osório FM Moraes LE Castro ALC Barros GML Alves RRN Fisheries and trade of seahorses in Brazil: historical perspective, current trends, and future directions Biodiversity and Conservation in press

[B232] Andrade J Folclore na região do Pará: teredos na alimentação/profissões ribeirinhas 1983 1 São Paulo: Escola de Folclore

[B233] Chaves FM Vilas Boas JC Anjos-Aquino EAC Perrelli MAdS, Albuquerque LBd, Anjos-Aquino EACd A utilização dos Mollusca pelos índios bororo: uma análise do acervo etnográfico do Museu Dom Bosco Descobrindo o museu: experiências de pesquisas e extensão no Museu Dom Bosco 2005 1 Campo Grande: Editora UCDB 161 166

[B234] Souza RM Alves AGC Alves MS Conhecimento sobre o molusco gigante africano Achatina fulica entre estudantes de uma escola pública na Região Metropolitana do Recife Revista Biotemas 2007 20 81 89

[B235] Nishida AK Nordi N Alves RRN Abordagem Etnoecológica da coleta de moluscos no Litoral Paraibano Tropical Oceanography 2004 32 53 68

[B236] Nishida AK Nordi N Alves RRN Molluscs production associated to lunar-tide cycle: a case study in Paraíba State under ethnoecology viewpoint Journal of Ethnobiology and Ethnomedicine 2006 2 6 10.1186/1746-4269-2-6 16784528 PMC1513198

[B237] Nishida AK Nordi N Alves RRdN Mollusc Gathering in Northeast Brazil: An Ethnoecological Approach Human Ecology 2006 34 133 145 10.1007/s10745-005-9005-x

[B238] Souto FJB Martins VS Conhecimentos etnoecológicos na mariscagem de moluscos bivalves no Manguezal do Distrito de Acupe, Santo Amaro-BA Revista Biotemas 2009 22 4 207 218

[B239] Martins VS Souto FJB Uma Análise biométrica de bivalves coletados por marisqueiras no manguezal de Acupe, Santo Amaro, Bahia: uma abordagem etnoconservacionista Sitientibus Série Ciências Biológicas 2006 6 98 10521774140

[B240] Martins VS Schiavetti A Souto FJB Ethnoecological knowledge of the artisan fishermen of octopi (Octopus spp.) in the community of Coroa Vermelha (Santa Cruz Cabrália, Bahia) Anais da Academia Brasileira de Ciências 2011 83 513 522 10.1590/s0001-37652011000200011 21670875

[B241] Dias TLP Leo Neto NA Alves RRN Molluscs in the marine curio and souvenir trade in NE Brazil: species composition and implications for their conservation and management Biodiversity and Conservation in press

[B242] Nomura H Os moluscos no folclore 2001 Mossoró, RN: Fundação Guimarães Duque e Fundação Vingt-un Rosado

[B243] Silva VMF Best RC Freshwater dolphin/fisheries interaction in the Central Amazon Amazoniana 1996 14 165 175

[B244] Costa-Neto EM Funch LS, Funch RR, Queiroz LP Os mamíferos da Chapada Diamantina e o conhecimento tradicional associado Serra do Sincorá: Parque Nacional da Chapada Diamantina 2008 Feira de Santana, BA, Brazil: RADAMI 201 21120422659

[B245] Estrela AR Costa-Neto EM, Santos-Fita D, Clavijo MV Etnoprimatologia y su aplicación en los planes de conservación de la Caatinga brasilera Manual de Etnozoología: una guía teórica-práctica para investigar la interconexión del ser humano con los animales 2009 1 Valencia, Spain: Tundra Ediciones21768914

[B246] Sabbatini G Stammati M Tavares MCH Giuliani MV Visalberghi E Interactions between humans and capuchin monkeys (Cebus libidinosus) in the Parque Nacional de Brasília, Brazil Applied Animal Behaviour Science 2006 97 272 283 10.1016/j.applanim.2005.07.002

[B247] Ribeiro GC Schiavetti A Costa-Neto EM, Santos-Fita D, Clavijo MV Conocimiento, creencias y utilización de recursos mastofaunísticos por los pobladores de la región del Parque Estatal de la Sierra del Conduru, Bahia, Brasil Manual de Etnozoología: una guía teórica-práctica para investigar la interconexión del ser humano con los animales 2009 1 Valencia, Spain: Tundra Ediciones 224 24121768914

[B248] Pinheiro L Cremer MJ Etnoecologia e captura acidental de golfinhos (Cetacea: Pontoporiidae e Delphinidae) na Baía da Babitonga, Santa Catarina Desenvolvimento e Meio Ambiente 2003 8 69 75

[B249] Schiavetti A Alarcon DT Rossi-Santos MR, Reis MdSS As possibilidades de estudos de *Sotalia guianensis *(van Bénéden, 1864) através do conhecimento local do pescadores Pesquisa e Conservação de Sotalia guianensis 2008 Ilhéus, BA, Brazil: Editus 189 196

[B250] Oliveira F Monteiro-Filho ELA Diegues AC Relação entre pescadores e botos na região de Cananéia: olhar e percepção caiçara Enciclopédia caiçara Festas, lendas e mitos caiçaras Hucitec, São Paulo: Hucitec, 414p 2006 5 São Paulo: Hucitec, USP/NUPAUB/CEC 253 270

[B251] Esbérard CEL Morcego: uma vítima das superstições Ciência Hoje 1995 18 71 7221774139

[B252] Gehara M Ribeiro GC Bisaggio EL Andriolo A Conhecimento popular de moradores do entorno do Parque Estadual do Ibitipoca (MG, Brasil) sobre o gênero Mazama Rafinesque, 1817 (Cervidae) Sitientibus 2009 9 122 128

[B253] Nomura H Os mamíferos no folclore 1996 1 Mossoró, RN, Brazil: Fundação Vingt-Un Rosado17332935

[B254] Rocha-Mendes F Kuczach AM Conhecimentos tradicionais sobre a mastofauna da região do Cânion do Guartelá, Estado do Paraná, sul do Brasil Sitientibus Série Ciências Biológicas 2007 7 323 33321774140

[B255] Rocha-Mendes F Mikich SB Bianconi GV Pedro WA Mamíferos do município de Fênix, Paraná: etnozoologia e conservação Revista Brasileira de Zoologia 2005 22 991 1002 10.1590/S0101-81752005000400027

[B256] Alves RRN Campos BATP Toledo GAC Mourão JS Barboza RRD Souto WMS PA G, Correa LM Traditional uses and conservation of dolphins in Brazil Dolphins: Anatomy, Behavior, and Threats 2010 New York: Nova Science Publishers, Inc 183 195

[B257] Barros FB Pereira HM Vicente L Use and Knowledge of the Razor-billed Curassow *Pauxi tuberosa *(Spix, 1825) (Galliformes, Cracidae) by a Riverine Community of the Oriental Amazonia, Brazil Journal of Ethnobiology and Ethnomedicine 2011 7 1 30 10.1186/1746-4269-7-1 21194497 PMC3023789

[B258] Alves RRN Nogueira E Araujo H Brooks S Bird-keeping in the Caatinga, NE Brazil Human Ecology 2010 38 147 156 10.1007/s10745-009-9295-5

[B259] Araujo HFP Lucena RFP Mourão JS Prenúncio de chuvas pelas aves na percepção de moradores de comunidades rurais no município de Soledade-PB, Brasil Interciencia 2005 30 764 769

[B260] Rocha MSP Cavalcanti PCM Sousa RL Alves RRN Aspectos da comercialização ilegal de aves nas feiras livres de Campina Grande, Paraíba, Brasil Revista de Biologia e Ciências da Terra 2006 6 204 221

[B261] Santos IB Costa-Neto EM Estudo Etnoornitológico em uma região do semi-árido do Estado da Bahia Sitientibus Série Ciências Biológicas 2007 7 273 28821774140

[B262] Bezerra DMMSQ Araujo HFP Alves RRN The use of wild birds by rural communities in the semi-arid region of Rio Grande do Norte State, Brazil Bioremediation, Biodiversity and Bioavailability 2011

[B263] Saiki PTO Guido LFE Cunha AMO Etnoecologia, etnotaxonomia e valoração cultural de Psittacidae em distritos rurais do Triângulo Mineiro, Brasil Revista Brasileira de Ornitologia 2009 17 41 52

[B264] Almeida SM Franchin AG Marçal Júnior O Estudo Etnoornitológico no Distrito Rural de Florentina, Município de Araguari, Região do Triângulo Mineiro, Minas Gerais Sitientibus Série Ciências Biológicas 2006 6 26 3621774140

[B265] Farias GB Alves AGC Albuquerque UP, Alves AGC, Araújo HFP Conhecimento prévio sobre a avifauna por alunos do Ensino Fundamental numa escola pública na Região Metropolitana do Recife: em busca de uma prática pedagógica culturalmente apropriada Povos e paisagens: etnobiologia, etnoecologia e biodiversidade no Brasil 2007 Recife: NUPPEA/UFRPE 48 59

[B266] Farias GB Alves ÂGC Nomenclatura e classificação etnoornitológica em fragmentos de Mata Atlântica em Igarassu, Região Metropolitana do Recife, Pernambuco Revista Brasileira de Ornitologia 2007 15 358 366

[B267] Araujo HFP Nishida AK Conhecimentos de pescadores artesanais sobre a avifauna em estuários paraibanos: uma contribuição para a conservação Sitientibus Série Ciências Biológicas 2007 7 67 7721774140

[B268] Farias GB Alves ÂGC Aves de Pernambuco: o estado atual do conhecimento ornitológico Biotemas 2009 22 1 10

[B269] Costa Neto EM As corujas e o homem: importância ecológica e relações culturais Ciência Hoje 1999 26 74 7621774139

[B270] Farias GBd Alves ÂGC É importante pesquisar o nome local das aves? Revista Brasileira de Ornitologia 2007 15 403 408

[B271] Farias GB Alves ÂGC Aspectos históricos e conceituais da etnoornitologia Biotemas 2007 20 91 100

[B272] Marques JGW Do canto bonito ao berro do bode: percepção do comportamento de vocalização em aves entre camponeses alagoanos Revista de Etologia 1999 special 71 85

[B273] Marques JGW Albuquerque UP, Alves AGC, Silva ACBL, Silva VA O sinal das aves. Uma tipologia sugestiva para uma etnoecologia com bases semióticas Atualidades em Etnobiologia e Etnoecologia 2002 1 1 Recife, PE, Brazil: SBEE 87 96

[B274] Nomura H Avifauna no folclore 1996 1 Mossoró, RN, Brazil: Fundação Vingt-un Rosado

[B275] Teixeira DM Perspectivas da etno-ornitologia no Brasil: o exemplo de um estudo sobre a "tapiragem" Boletim do Museu Paraense Emílio Göeldi 1992 8 113 121

[B276] Fernandes-Ferreira H Mendonça SV Albano C Ferreira FS Alves RRN Alves RRN, Souto WMS, Mourão JS Comércio e criação de aves silvestres (Psittaciformes, Piciformes e Passeriformes) no Estado do Ceará A Etnozoologia no Brasil: Importância, Status atual e Perspectivas 2010 7 1 Recife, PE, Brazil: NUPEEA 379 40221461541

[B277] Almeida MB Carneiro da Cunha M Smith M Carneiro da Cunha M, Almeida MB Classificação dos animais da Reserva Extrativista do Alto Juruá pelos Seringueiros Enciclopédia da Floresta o O Alto Juruá: práticas e conhecimentos das populações 2002 Companhia das Letras 419 429

[B278] Posey DA Hierarchy and utility in a folk taxonomic system: patterns in classification of arthropods by the Kaypó Indians of Brazil Journal of Ethnobiology 1984 4 123 139

[B279] Costa-Neto EM Folk Taxonomy and Cultural Significance of "Abeia" (Insecta, Hymenoptera) to the Pankarare, Northeastern Bahia State, Brazil Journal of Ethnobiology 1998 18 1 13

[B280] Mourão JS Araujo HFP Almeida FS Ethnotaxonomy of mastofauna as practised by hunters of the municipality of Paulista, state of Paraíba-Brazil Journal of Ethnobiology and Ethnomedicine 2006 2 7 10.1186/1746-4269-2-7 16603080 PMC1473038

[B281] Souza SP Begossi A Whales, dolphins or fishes? The ethnotaxonomy of cetaceans in São Sebastião, Brazil Journal of Ethnobiology and Ethnomedicine 2007 3 1 9 10.1186/1746-4269-3-1 17311681 PMC1804260

[B282] Calo CFF Schiavetti A Cetra M Local ecological and taxonomic knowledge of snapper fish Actinopterygii:Teleostei) held by fishermen in Ilhéus, Bahia, Brazil Neotropical Ichthyology (Impresso) 2009 7 403 414 10.1590/S1679-62252009000300007

[B283] Marques JGW Pose LM Taxonomia e etnotaxonomia dos mugilídeos do Complexo Estuarino-Lagunar Mundaú-Manguaba, AL. Aspectos Morfológicos Ciência e Cultura 1990 42 532 53321774139

[B284] Mourão JS Montenegro SCS Pescadores e Peixes: O conhecimento local e o uso da taxonomia folk baseada no modelo berliniano 2006 2 Recife, PE, Brazil: Editora Livro Rápido

[B285] Mourão JS Nordi N Comparações entre as Taxonomias Folk e Científica para peixes do Estuário do Rio Mamanguape, Paraíba-Brasil Interciencia 2002 27 664 668

[B286] Mourão JS Nordi N Principais critérios utilizados por pescadores artesanais na taxonomia folk dos peixes do estuário do rio Mamanguape, Paraíba-Brasil Interciencia 2002 27 607 612

[B287] Ferreira EN Mourão JS Rocha PD Nascimento DM Bezerra DMMSQ Folk classification of the crabs and swimming crabs (Crustacea - Brachyura) of the Mamanguape river estuary, Northeastern - Brazil Journal of Ethnobiology 2009 5 1 11 10.1186/1746-4269-5-22 PMC2734541 19671153

[B288] Silva GO Tudo que tem na terra tem no mar. A classificação dos seres vivos entre os trabalhadores da pesca em Piratininga, Rio de Janeiro 1988 1 Rio de Janeiro, RJ, Brazil: FUNARTE/Instituto Nacional do Folclore

[B289] Ferreira EN Mourão JS Rocha PD Nascimento DM Bezerra DMMQS Alves RRN, Souto WMS, Mourão JS Classificação etnobiológica de caranguejos e siris (CRUSTACEA - BRACHYURA) do estuário do rio Mamanguape, Paraíba - Brasil A Etnozoologia no Brasil: Importância, Status atual e Perspectivas 2010 7 1 Recife, PE, Brazil: NUPEEA 211 23221461541

[B290] Begossi A Clauzet M Figueiredo JL Guarano L Lima R Lopes PFM Souza MR Silva AL Silvano RAM Are biological species and high-ranking categories real? Fish folk taxonomy in the Atlantic Forest and the Amazon (Brazil) Current Anthropology 2008 49 291 302 10.1086/527437

[B291] Costa-Neto EM Ethnotaxonomy and use of bees in Northeastern Brazil The Food Insects Newsletter 1996 9 1 3

[B292] Teixeira DM Papavero N Kury LB As aves do Pará segundo as "Memórias" de Dom Lourenço Álvares Roxo de Potflis (1752) Arquivos de Zoologia 2010 41 97 131

[B293] Papavero N Teixeira DM A fauna da Amazônia brasileira nos relatos de viajantes e cronistas nos séculos XVI a XVIII. 1. O descobrimento da foz do rio Amazonas por Pinzón (1500) e a primeira citação de um animal brasileiro Contribuições Avulsas sobre a História Natural do Brasil 1999 7 1 2

[B294] Papavero N Teixeira DM Luz JRP A fauna da Amazônia brasileira nos relatos de viajantes e cronistas dos séculos XVI a XVIII. 3. A relação de Francisco Vásquez sobre a viagem de Pedro de Ursúa e Lope de Aguirre (1559-1561) pelo Amazonas Contribuições Avulsas sobre a História Natural do Brasil 1999 9 1 5

[B295] Papavero N Teixeira DM Luz JRP A fauna da Amazônia brasileira nos relatos de viajantes e cronistas dos séculos XVI a XVIII. 4. Notas miscelâneas sobre relatos curtos dos séculos XVII e XVIII Contribuições Avulsas sobre a História Natural do Brasil 1999 10 1 4

[B296] Papavero N Teixeira DM Luz JRP A fauna da Amazônia brasileira nos relatos de viajantes e cronistas dos séculos XVI a XVIII. 5. Symão Estacio da Sylveira e o Intento da Jornada do Pará (1618) Contribuições Avulsas sobre a História Natural do Brasil 1999 11 1 7

[B297] Papavero N Teixeira DM Luz JRP A fauna da Amazônia brasileira nos relatos de viajantes e cronistas dos séculos XVI a XVIII. 6. A Relação sumaria das cousas do Maranhão de Symão Estacio da Sylveira (1624) Contribuições Avulsas sobre a História Natural do Brasil 1999 12 1 10

[B298] Papavero N Teixeira DM Luz JRP A fauna da Amazônia brasileira nos relatos de viajantes e cronistas dos séculos XVI a XVIII. 7. John Day e A Publication of Guiana's plantation (1632) Contribuições Avulsas sobre a História Natural do Brasil 1999 12 1 12

[B299] Papavero N Teixeira DM Luz JRP A fauna da Amazônia brasileira nos relatos de viajantes e cronistas dos séculos XVI a XVIII. 8. A viagem de Pedro Teixeira (1637-1639) Contribuições Avulsas sobre a História Natural do Brasil 1999 14 1 5

[B300] Papavero N Teixeira DM Luz JRP A fauna da Amazônia brasileira nos relatos de viajantes e cronistas dos séculos XVI a XVIII. 9. A Relación del descubrimiento del Rio de las Amazonas do Pe. Alonso de Rojas (?1639) Contribuições Avulsas sobre a História Natural do Brasil 1999 15 1 7

[B301] Papavero N Teixeira DM Luz JRP A fauna da Amazônia brasileira nos relatos de viajantes e cronistas dos séculos XVI a XVIII. 10. O Padre Cristóbal de Acuña (1641) Contribuições Avulsas sobre a História Natural do Brasil 1999 16 1 4

[B302] Papavero N Teixeira DM Luz JRP A fauna da Amazônia brasileira nos relatos de viajantes e cronistas dos séculos XVI a XVIII. 11. A Descrição do Estado do Maranhão, Pará, Corupá e Rio das Amazonas de Maurício de Heriarte (1662) Contribuições Avulsas sobre a História Natural do Brasil 1999 17 1 3

[B303] Papavero N Teixeira DM Luz JRP A fauna da Amazônia brasileira nos relatos de viajantes e cronistas dos séculos XVI a XVIII. 12. A viagem de la Condamine (1743) Contribuições Avulsas sobre a História Natural do Brasil 1999 18 1 7

[B304] Papavero N Teixeira DM Luz JRP A fauna da Amazônia brasileira nos relatos de viajantes e cronistas dos séculos XVI a XVIII. 13. Dom João de São Joseph Queiroz, Bispo do Grão-Pará (1761-1763) Contribuições Avulsas sobre a História Natural do Brasil 1999 19 1 2

[B305] Papavero N Teixeira DM Luz JRP A fauna da Amazônia brasileira nos relatos de viajantes e cronistas dos séculos XVI a XVIII. 14.Francisco Xavier Ribeiro de Sampaio e a primeira relação da fauna do Rio Branco (1774-1775) Contribuições Avulsas sobre a História Natural do Brasil 1999 20 1 9

[B306] Papavero N Teixeira DM Luz JRP A fauna da Amazônia brasileira nos relatos de viajantes e cronistas dos séculos XVI a XVIII. 15. O Tesouro descoberto no Rio Amazonas do Pe. João Daniel (1758-1776) Contribuições Avulsas sobre a História Natural do Brasil 1999 21 1 7

[B307] Papavero N Teixeira DM A fauna de Campos dos Goytacazes, Província do Rio de Janeiro, em 1881, segundo José Alexandre Teixeira de Mello Contribuições Avulsas sobre a História Natural do Brasil 2000 33 1 2

[B308] Carrera M A entomologia na história natural de Plínio Revista Brasileira de Entomologia 1993 37 387 396

[B309] Carrera M Escarabeídeos fúnebres e sagrados Revista Brasileira de Entomologia 1995 39 475 477

[B310] Galvão E O cavalo na América indígena: nota prévia a um estudo de mudança cultural Revista do Museu Paulista 1963 14 222 232

[B311] Gilmore R Ribeiro D Fauna e etnozoologia da América do Sul tropical Suma Etnológica Brasileira Etnobiologia 1986 Petrópolis, RJ, Brazil: Vozes/Finep 189 23321701765

[B312] Papavero N A "Memoria Acêrca das Abêlhas da Provincia do Piauhí, no Imperio do Brazil" de Leonardo da Senhora das Dores Castello-Branco, (1842), segundo o autógrafo do autor no Instituto Histórico e Geográfico Brasileiro, Rio de Janeiro. I. Introdução e texto Contribuições Avulsas sobre a História Natural do Brasil 1999 3 1 10

[B313] Papavero N Teixeira DM Navegação do Rio Tietê, na Província de São Paulo, até o Rio Taquari, na Província do Mato Grosso, por Francisco de Oliveira Barbosa (1792), com comentários sobre a fauna Contribuições Avulsas sobre a História Natural do Brasil 2000 36 1 3

[B314] Papavero N Teixeira DM A fauna brasileira do "Vocabulario na lingua brasilica" de Leonardo do Valle, S. J. (1585) Contribuições Avulsas sobre a História Natural do Brasil 1999 1 1 8

[B315] Papavero N Teixeira DM A fauna de São Paulo nos séculos XVI a XVIII nos textos de viajantes, cronistas, missionários e relatos monçoeiros 2007 São Paulo, SP, Brazil: Editora da Universidade de São Paulo

[B316] Papavero N Teixeira DM Braz da Costa Rubim e sua lista de animais da Província do Espírito Santo Contribuições Avulsas sobre a História Natural do Brasil 2000 31 1 2

[B317] Papavero N Teixeira DM Overal WL Notas sobre a história da zoologia do Brasil. 2. As viagens de Francisco de Melo Palheta, o introdutor do cafeeiro no Brasil Boletim do Museu Paraense Emilio Goeldi 2002 17 181 207

[B318] Papavero N Teixeira DM A primeira menção de animais para a Capitania do Espírito Santo, no Brasil, pelo Pe. Afonso Braz, S. J. (1551) Contribuições Avulsas sobre a História Natural do Brasil 2000 27 1 2

[B319] Papavero N Teixeira DM Figueiredo JL Pujol-Luz JR Os capítulos sobre animais dos "Dialogos Geograficos, Chronologicos, Politicos, e Naturales" (1769) de Joseph Barboza de Sáa e a primeira monografia sobre a fauna de Mato Grosso Arquivos de Zoologia 2009 40 75 154

[B320] Souza RF Medicina e fauna silvestre em Minas Gerais no século XVIII Varia Historia 2008 24 273 291

[B321] Teixeira DM Llorente-Bousquets J Papavero N Animales de la Alegoría Brasileña - Ferdinand van Kessel, 1648-1697 Ciencia y Desarrollo 1997 131 36 44

[B322] Teixeira DM Papavero N Monne MA Insetos em presépios e as "formigas vestidas" de Jules Martin (1832-1906): Uma curiosa manufatura paulista do final do século XIX Anais do Museu Paulista 2008 16 105 127 10.1590/S0101-47142008000200004

[B323] Teixeira DM Papavero N Os animais do descobrimento: A fauna brasileira mencionada nos documentos relativos à viagem de Pedro Álvares Cabral (1500-1501) Publicações Avulsas do Museu Nacional 2006 1 13621771129

[B324] Nomura H História da Zoologia no Brasil - século XVII 1996 Mossoró, RN: Fundação Vingt-un Rosado e ETFRN-UNED21766508

[B325] Nomura H História da Zoologia no Brasil - século XVI 1996 Mossoró, RN: FundaçãoVingt-un Rosado e ETFRN-UNED21766508

[B326] Nomura H História da Zoologia no Brasil - século XVIII 1998 Lisboa, Portugal: Museu Bocage - Museu Nacional de História Natural21766508

[B327] Miranda EE O descobrimento da biodiversidade. A ecologia de índios, jesuítas e leigos no século XVI 2004 1 São Paulo, SP: Ed Loyola

[B328] Vanzolini PE A contribuição zoológica dos primeiros naturalistas viajantes no Brasil Dossiê Viajantes do Brasil, Revista USP 1996 30 90 23821772408

[B329] Alves RRN Commercialization of Uranoscodon superciliosus Linnaeus, 1758 (Tropiduridae) for magical-religious purposes in North and Northeastern of Brazil Sitientibus Série Ciências Biológicas 2008 8 257 25821774140

[B330] Cravalho MA Shameless creatures: An ethnozoology of the Amazon River dolphin Ethnology 1999 38 47 58 10.2307/3774086

[B331] Posey DA Elisabetsky E Conceito de animais e seus espíritos em relação a doenças e curas entre os índios Kayapó da Aldeia Gorotire, Pará Boletim do Museu Paraense Emílio Goeldi 1991 7 21 36

[B332] Costa-Neto EM "Caçando" bichos na selva urbana: um estudo de caso na cidade de Feira de Santana, Bahia, Brasil Bioikos 2004 18 21 25

[B333] Léo Neto NA Brooks SE Alves RRN From Eshu to Obatala: animals used in sacrificial rituals at Candomble "terreiros" in Brazil Journal of Ethnobiology and Ethnomedicine 2009 5 1 23 10.1186/1746-4269-5-1 19709402 PMC2739163

[B334] Léo Neto NA Alves RRN A Natureza Sagrada do Candomblé: Análise da construção mística acerca da natureza em terreiros de Candomblé em Caruaru (PE) e Campina Grande (PB) Interciencia 2010 35 568 574

[B335] Léo Neto NA Alves RRN Alves RRN, Souto WMS, Mourão JS "Sangue e música": animais utilizados em rituais de sacrifício em terreiros de Candomblé A Etnozoologia no Brasil: Importância, Status atual e Perspectivas 2010 7 1 Recife, PE, Brazil: NUPEEA 495 51221461541

[B336] Da Matta R Soárez E Águias, burros e borboletas: um estudo antropológico do jogo do bicho 1999 1 Rio de Janeiro, Brazil: Rocco

[B337] Marques JGW Alves AGC, Lucena RFP, Albuquerque UP É Pecado Matar a Esperança, mas Todo Mundo quer Matar o Sariguê. Etnoconservação e Catolicismo Popular no Brasil Atualidades em Etnobiologia e Etnoecologia 2005 Recife, PE, Brazil: NUPPEA 27 43

[B338] Marques JGW Pedrosa TM Etnoecologia e Ornitomancia Macabra. Aves Alagoanas, Gente Marcada para Morrer & Mortes Anunciadas Arte popular de Alagoas 2000 Macéio, AL, Brazil: UFAL 97 100

[B339] Nomura H Usos, crendices e lendas sobre peixes 1996 1 Mossoró, RN, Brazil: Fundação Vingt-Un Rosado

[B340] Teixeira DM FUNARTE/Instituto Nacional do Folclore Um estudo de etnozoologia Karajá: o exemplo das máscaras de Aruanã O artesão tradicional e seu papel na sociedade contemporânea 1983 Rio de Janeiro, Brazil: FUNARTE/Instituto Nacional do Folclore21743596

[B341] Farias GB Alves AGC Marques JGW Mythological Relations Between the "Lavandeira" Birds *Fluvicola nengeta *and *Motacilla alba *in Northeast Brazil and Northwest Spain: Possible Cultural Implications for Conservation Journal of Ethnobiology 2010 30 240 251 10.2993/0278-0771-30.2.240

[B342] Marques JGW "Pássaro" É Bom para se Pensar:Simbolismo Ascensional em uma Etnoecologia do Imaginário Incelências Revista do Núcleo de Programas Pesquisa 2010 1 6 2721396934

[B343] Rosa IML Oliveira TPR Alves RRN Alves RRN, Souto WMS, Mourão JS Entre o corpo e o espírito: uso medicinal e mágico-religioso de cavalos-marinhos no Brasil A Etnozoologia no Brasil: Importância, Status atual e Perspectivas 2010 7 1 Recife, PE, Brazil: NUPEEA 323 34621461541

[B344] Alves RRN Rosa IL Use of Tucuxi Dolphin Sotalia fluviatilis for Medicinal and Magic/Religious Purposes in North of Brazil Human Ecology 2008 36 443 447 10.1007/s10745-008-9174-5

[B345] Alves RRN Santana GG Use and commercialization of Podocnemis expansa (Schweiger 1812) (Testudines: Podocnemididae) for medicinal purposes in two communities in North of Brazil Journal of Ethnobiology and Ethnomedicine 2008 4 6 10.1186/1746-4269-4-6 18208597 PMC2254592

[B346] Branch L Silva MF Folk medicine in Alter do Chão, Pará, Brasil Acta Amazônica 1983 13 737 797

[B347] Costa RPC Silva WG Medicina popular da Amazônia brasileira I: identificação dos ácidos graxos e triglicerídeos da banha da cobra sucuriju (*Eunnects murinus*) Revista da Universidade do Amazonas (Série Ciências da Saúde) 1993 2 73 90

[B348] Figueiredo N Os 'bichos' que curam: os animais e a medicina 'folk' em Belém do Pará Boletim do Museu Paraense Emílio Göeldi 1994 10 75 91

[B349] Pinto AAC Maduro CB Produtos e subprodutos da medicina popular comercializados na cidade de Boa Vista, Roraima Acta Amazônica 2003 33 281 290

[B350] Silva AL Animais medicinais: conhecimento e uso entre as populações ribeirinhas do rio Negro, Amazonas, Brasil Boletim do Museu Paraense Emílio Göeldi 2008 3 343 357

[B351] Almeida CFCBR Albuquerque UP Uso e conservação de plantas e animais medicinais no Estado de Pernambuco (Nordeste do Brasil): Um estudo de caso Interciencia 2002 27 276 285

[B352] Alves RRN Soares TC Mourão JS Uso de animais medicinais na comunidade de Bom Sucesso, Soledade, Paraíba Sitientibus Série Ciências Biológicas 2008 8 142 14721774140

[B353] Alves RRN Barbosa JAA Santos SLDX Souto WMS Barboza RRD Animal-based Remedies as Complementary Medicines in the Semi-arid Region of Northeastern Brazil eCAM 2009 nep134 10.1093/ecam/nep134 PMC3094714 19729490

[B354] Alves RRN Lima HN Tavares MC Souto WMS Barboza RRD Vasconcellos A Animal-based remedies as complementary medicines in Santa Cruz do Capibaribe, Brazil BMC Complementary and Alternative Medicine 2008 8 44 10.1186/1472-6882-8-44 18647413 PMC2503950

[B355] Alves RRN Oliveira MGG Barboza RRD Singh R Lopez LLC Medicinal Animals as Therapeutic Alternative in a Semi-Arid Region of Northeastern Brazil Research in Complementary Medicine 2009 16 305 312 19887809 10.1159/000235855

[B356] Alves RRN Oliveira MdGG Barboza RRD Lopez LCS An ethnozoological survey of medicinal animals commercialized in the markets of Campina Grande, NE Brazil Human Ecology Review 2010 17 11 17

[B357] Andrade JN Costa-Neto EM O comércio de produtos zooterápicos na cidade de Feira de Santana, Bahia, Brasil Sitientibus 2006 6 37 43

[B358] Andrade JN Costa-Neto EM Primeiro registro da utilização medicinal de recursos pesqueiros na cidade de São Félix, Estado da Bahia, Brasil Acta Sci Biol Sci 2005 27 177 183

[B359] Barboza RRD Souto WMS Mourão JS The use of zootherapeutics in folk veterinary medicine in the district of Cubati, Paraíba State, Brazil Journal of Ethnobiology and Ethnomedicine 2007 3 14 10.1186/1746-4269-3-14 17825094 PMC2008192

[B360] Confessor M Mendonca L Mourao J Alves R Animals to heal animals: ethnoveterinary practices in semi-arid region, Northeastern Brazil Journal of Ethnobiology and Ethnomedicine 2009 5 37 10.1186/1746-4269-5-37 19941663 PMC2788532

[B361] Costa-Neto EM A zooterapia popular no Estado da Bahia: registro de novas espécies animais utilizadas como recursos medicinais Ciência & Saúde Coletiva 2011 16 1639 1650 21503516 10.1590/s1413-81232011000700100

[B362] Costa-Neto EM Conhecimento e usos tradicionais de recursos faunísticos por uma comunidade Afro-Brasileira. Resultados preliminares Interciencia 2000 25 423 431

[B363] Costa-Neto EM The Use of Insects in Folk Medicine in the State of Bahia, Northeastern Brazil, With Notes on Insects Reported Elsewhere in Brazilian Folk Medicine Human Ecology 2002 30 245 263 10.1023/A:1015696830997

[B364] Costa-Neto EM Oliveira MVM Cockroach is Good for Asthma: Zootherapeutic Practices in Northeastern Brazil Human Ecology Review 2000 7 41 51

[B365] Costa-Neto EM Pacheco JM Utilização medicinal de insetos no povoado de Pedra Branca, Santa Terezinha, Bahia, Brasil Biotemas 2005 18 113 133

[B366] Costa-Neto EM Faunistc Resources used as medicines by an Afro-brazilian community from Chapada Diamantina National Park, State of Bahia-Brazil Sitientibus 1996 211 219

[B367] Costa-Neto EM Healing with animals in Feira de Santana City, Bahia, Brazil Journal of Ethnopharmacology 1999 65 225 230 10.1016/S0378-8741(98)00158-5 10404420

[B368] Costa-Neto EM Recursos animais utilizados na medicina tradicional dos índios Pankararé que habitam o Nordeste do Estado da Bahia, Brasil Actualidades Biologicas 1999 21 69 79

[B369] Costa-Neto EM Traditional use and sale of animals as medicines in Feira de Santana City, Bahia, Brazil Indigenous Knowledge and Development Monitor 1999 7 6 9

[B370] Ferreira FS Brito S Ribeiro S Saraiva A Almeida W Alves RRN Animal-based folk remedies sold in public markets in Crato and Juazeiro do Norte, Ceara, Brazil BMC Complementary and Alternative Medicine 2009 9 17 10.1186/1472-6882-9-17 19493345 PMC2698880

[B371] Ferreira FS Brito S Ribeiro S Almeida W Alves RRN Zootherapeutics utilized by residents of the community Poco Dantas, Crato-CE, Brazil Journal of Ethnobiology and Ethnomedicine 2009 5 21 10.1186/1746-4269-5-21 19656376 PMC2731045

[B372] Moura FBP Marques JGW Zooterapia popular na Chapada Diamantina: uma Medicina incidental? Ciência & Saúde Coletiva 2008 13 2179 2188 19039402 10.1590/s1413-81232008000900023

[B373] Souto WMS Mourão JS Barboza RRD Rocha MSP Alves RRN Animal-based medicines used in ethnoveterinary practices in the semi-arid region of Northeastern Brazil Anais da Academia Brasileira de Ciências 2010 in press 10.1590/s0001-37652012005000038 22751649

[B374] Barbosa JAA Alves RRN "Um chá de que?" - Animais Utilizados no Preparo tradicional de Bebidas Medicinais no Agreste Paraibano BioFar 2010 4 2 1 12

[B375] Barboza RRD Alves RRN Souto WMS Mourão JS Costa-Neto EM, Alves RRN Etnoveterinária: o conhecimento milenar que cura e trata os animais Zooterapia: Os Animais na Medicina Popular Brasileira 2010 2 1 Recife, PE, Brazil: NUPEEA 103 124

[B376] Silva NLG Ferreira FS Coutinho HDM Alves RRN Costa-Neto EM, Alves RRN Zooterápicos utilizados em comunidades rurais do município de Sumé, semiárido da Paraíba, Nordeste do Brasil Zooterapia: Os Animais na Medicina Popular Brasileira 2010 2 1 Recife, PE, Brazil: NUPEEA 243 267

[B377] Souto WMS Alves RRN Confessor MVA Barboza RRD Mourão JS Mendonça LET Alves RRN, Souto WMS, Mourão JS A Zooterapia na Etnoveterinária do semi-árido paraibano A Etnozoologia no Brasil: Importância, Status atual e Perspectivas 2010 7 1 Recife, PE, Brazil: NUPEEA 423 44621461541

[B378] Souto WMS Mourão JS Barboza RRD Alves RRN Parallels between zootherapeutic practices in Ethnoveterinary and Human Complementary Medicine in NE Brazil Journal of Ethnopharmacology 2011 134 3 753 767 10.1016/j.jep.2011.01.041 21291986

[B379] Costa-Neto EM Animal Species Traded as Ethnomedicinal Resources in the Federal District, Central West Region of Brazil The Open Complementary Medicine Journal 2010 2 24 30 10.2174/1876391X01002020024

[B380] Oliveira ES Torres DF Brooks SE Alves RRN The medicinal animal markets in the metropolitan region of Natal City, Northeastern Brazil Journal of Ethnopharmacology 2010 130 1 54 60 10.1016/j.jep.2010.04.010 20460145

[B381] Pessoa RS Almeida AV Alves ÂGC Melo LEH A "maçã-do-boi" (Bezoário): Etnomedicina, História e Ciência Sitientibus 2002 2 55 61

[B382] Silva MLVd Alves ÂGC Almeida AVd A zooterapia no Recife (Pernambuco): uma articulação entre as práticas e a história Biotemas 2004 17 95 116

[B383] Alves RRN Use of Marine Turtles in Zootherapy in Northeast Brazil Marine Turtle Newsletter 2006 112 16 17

[B384] Alves RRN Filho GAP Lima YCC Snakes used in Ethnomedicine in Northeast Brazil Environment, Development and Sustainability 2006 9 455 464

[B385] Alves RRN Rosa IL Zootherapeutic practices among fishing communities in North and Northeast Brazil: A comparison Journal of Ethnopharmacology 2007 111 82 103 10.1016/j.jep.2006.10.033 17118592

[B386] Alves RRN Rosa IL From cnidarians to mammals: The use of animals as remedies in fishing communities in NE Brazil Journal of Ethnopharmacology 2006 107 259 276 10.1016/j.jep.2006.03.007 16621379

[B387] Begossi A Food taboos at Búzios Island (Brazil): their significance and relation to folk medicine Journal of Ethnobiology 1992 12 117 139

[B388] Costa-Neto EM Marques JGW Faunistic resources used as medicines by artisanal fishermen from Siribinha Beach, State of Bahia, Brazil Journal of Ethnobiology 2000 20 93 109

[B389] Alves RRN Fauna used in popular medicine in Northeast Brazil Journal of Ethnobiology and Ethnomedicine 2009 5 1 30 10.1186/1746-4269-5-1 19128461 PMC2628872

[B390] Alves RRN Pereira Filho GA Commercialization and use of snakes in North and Northeastern Brazil: implications for conservation and management Biodivers Conserv 2007 16 969 985 10.1007/s10531-006-9036-7

[B391] Alves RRN Pereira Filho GA Hawksworth DL, Bull AT Commercialization and use of snakes in North and Northeastern Brazil: implications for conservation and management Vertebrate Conservation and Biodiversity 2007 1 Amsterdan: Springer Netherlands 143 159

[B392] Begossi A Hanazaki N Ramos R Pieroni A, Price L Healthy fish: medicinal and recommended species in the Amazon and in the Atlantic Forest coast (Brazil) Eating and Healing, traditional food as medicine 2006 1 New York: The Haworth Press 237 250

[B393] Alves RRN Barboza RRD Souto MSW Mourão JS Utilization of Bovids in traditional folk medicine and their implications for conservation Environmental Research Journal 2011 5 547 562

[B394] Alves RRN Rosa IL Medicinal animals for the treatment of asthma in Brazil BMC Complementary and Alternative Medicine 2008 14 350 351 10.1089/acm.2008.0032 18435600

[B395] Alves RRN Rosa IL Santana GG The Role of Animal-derived Remedies as Complementary Medicine in Brazil BioScience 2007 57 949 955 10.1641/B571107

[B396] Alves RRN Silva CC Barboza RRD Souto WMS Zootherapy as an alternative therapeutic in South America Journal of Alternative Medicine Research 2009 1 21 47

[B397] Alves RRN Silva CC Barboza RRD Souto WMS Levine JK Zootherapy as alternative therapeutic in South America Low Incomes: Social, Health and Educational Impacts 2009 New York, USA: Nova Science Publishers

[B398] Alves RRN Souto WMS Barboza RRD Primates in traditional folk medicine: a world overview Mammal Review 2010 40 155 180 10.1111/j.1365-2907.2010.00158.x

[B399] Alves RRN Vieira WLS Santana GG Reptiles used in traditional folk medicine: conservation implications Biodiversity and Conservation 2008 17 8 2037 2049 10.1007/s10531-007-9305-0

[B400] Alves RRN Costa-Neto EM, Santos-Fita D, Clavijo MV Zooterapia: importancia, usos e implicaciones conservacionistas Manual de Etnozoología: una guía teórica-práctica para investigar la interconexión del ser humano con los animales 2009 1 Valencia, Spain: Tundra Ediciones21768914

[B401] Alves RRN Neto NAL Brooks SE Albuquerque UP Commercialization of animal-derived remedies as complementary medicine in the semi-arid region of Northeastern Brazil Journal of Ethnopharmacology 2009 124 600 608 10.1016/j.jep.2009.04.049 19422902

[B402] Carrera M Terapêutica entomológica Revista Brasileira de Entomologia 1993 37 193 198

[B403] Costa-Neto EM Implications and applications of folk zootherapy in the state of Bahia, Northeastern Brazil Sustainable Development 2004 12 161 174 10.1002/sd.234

[B404] Costa-Neto EM Etnoentomologia alagoana, com ênfase na utilização medicinal de insetos 1994 Macéio, AL, Brasil: Centro de Ciências Biológicas, Universidade Federal de Alagoas21774136

[B405] Costa-Neto EM Barata é um santo remédio: introdução à zooterapia popular no estado da Bahia 1999 1 Feira de Santana, Brazil: EdUEFS21770179

[B406] Costa-Neto EM Honey bees from Brazil: diversity of insect-product used by the Pankararé Honey Bee 1999 10 17 18

[B407] Francis DG Equoterapia: recurso inovador para reabilitação física e mental Anais de Etologia 1996 14 59 63

[B408] Aguiar JH Francis DG Equoterapia. Recurso inovador para reabilitacao fisica e mental Veterinaria Noticias (Brazil) 1998 4 130 134

[B409] Marques JG Costa-Neto EM Insects as folk medicines in the State of Alagoas, Brazil The Food Inse News 1997 10 7 10

[B410] Marques JGW Costa-Neto EM Insect cure for ailments Honey Bee 1999 10 1 17

[B411] Marques JGW Insects as folk medicines in the State of Alagoas, Brazil Insect Food Newsletter 1995 1 75

[B412] Marques JGW Fauna medicinal: Recurso do ambiente ou ameaça à biodiversidade? Mutum 1997 1 4

[B413] Nogueira D As qualidades ocultas dos crustáceos Ciência Hoje 1999 25 4721774139

[B414] Souto WMS Barboza RRD Mourão JS Alves RRN Zootherapy in Brazil: An Urgent Necessity of Interdisciplinary Studies West Indian Medical Journal 2009 58 494 495 20443230

[B415] Almeida AV Alves AGC, Lucena RFP, Albuquerque UP Prescrições zooterápicas indígenas brasileiras nas obras de Guilherme Piso (1611-1679) Atualidades em Etnobiologia e Etnoecologia 2005 1 Recife, Brazil: Sociedade Brasileira de Etnobiologia e Etnoecologia, Nuppea 47 60

[B416] Alves RRN Rosa IL Trade of animals used in Brazilian traditional medicine: trends and implications for conservation Human Ecology 2010 38 691 704 10.1007/s10745-010-9352-0

[B417] Alves RRN Barboza RRD Souto WMS Columbus A, Kuznetsov L Endangered Felidae Used in Traditional Medicine Endangered Species: New Research 2009 Hauppauge, NY, EUA: Nova Science Publishers, Inc 343 356

[B418] Alves RRN Barboza RRD Souto WMS Endangered Felidae used in Traditional Medicines International Journal of Medical and Biological Frontiers 2009 15 357 370

[B419] Alves RRN Barboza RRD Souto WMS Mourão JS Kudrow NJ Utilization of Bovids in Traditional Folk Medicine and Their Implications for Conservation Conservation of Natural Resources 2009 Hauppauge, NY, EUA: Nova Science Publishers, Inc 191 206

[B420] Alves RRN Barboza RRD Souto WMS A Global overview of canids used in traditional medicines Biodiversity and Conservation 2010 19 1513 1522 10.1007/s10531-010-9805-1

[B421] Alves RRN Dias TLP Usos de invertebrados na medicina popular no Brasil e suas implicações para conservação Tropical Conservation Science 2010 3 159 174

[B422] Almeida AV Costa-Neto EM, Alves RRN A zooterapia adotada pelos médicos Simão Pinheiro Morão (c. 1618-1685) e João Ferreyra da Rosa (c. 1659-1725) em Pernambuco no final do século XVII Zooterapia: Os Animais na Medicina Popular Brasileira 2010 2 1 Recife, PE, Brazil: NUPEEA 55 74

[B423] Alves RRN Costa-Neto EM, Alves RRN O comércio de recursos zooterápicos Zooterapia: Os Animais na Medicina Popular Brasileira 2010 2 1 Recife, PE, Brazil: NUPEEA 159 176

[B424] Bomfim GF Costa-Neto EM Uetanabaro APT Costa-Neto EM, Alves RRN Cupinzeiros: utilização na medicina tradicional e avaliação da atividade antimicrobiana *in vitro* Zooterapia: Os Animais na Medicina Popular Brasileira 2010 2 1 Recife, PE, Brazil: NUPEEA 177 188

[B425] Chemas RC Costa-Neto EM, Alves RRN A zooterapia no âmbito da medicina civilizada. I. Organoterapia humana e animal stricto sensu Zooterapia: Os Animais na Medicina Popular Brasileira 2010 2 1 Recife, PE, Brazil: NUPEEA 75 102

[B426] Costa-Neto EM Alves RRN Zooterapia: Os Animais na Medicina Popular Brasileira 2010 1 Recife, PE, Brazil: NUPEEA

[B427] Costa-Neto EM Alves RRN Costa-Neto EM, Alves RRN Estado da arte da zooterapia popular no Brasil Zooterapia: Os Animais na Medicina Popular Brasileira 2010 2 1 Recife, PE, Brazil: NUPEEA 13 54

[B428] Alves RRN Léo Neto NA Santana GG Vieira WLS Almeida WO Reptiles used for medicinal and magic religious purposes in Brazil Applied Herpetology 2009 6 257 274 10.1163/157075409X432913

[B429] Oliva VNLS Costa-Neto EM, Alves RRN Terapia Assistida por Animais Zooterapia: Os Animais na Medicina Popular Brasileira 2010 2 1 Recife, PE, Brazil: NUPEEA 125 140

[B430] Ribeiro GC Pereira JPR Docio L Alarcon DT Schiavetti A Costa-Neto EM, Alves RRN Zooterápicos utilizados no sul da Bahia Zooterapia: Os Animais na Medicina Popular Brasileira 2010 2 1 Recife, PE, Brazil: NUPEEA 221 242

[B431] Alves RRN Alves HN The faunal drugstore: Animal-based remedies used in traditional medicines in Latin America Journal of Ethnobiology and Ethnomedicine 2011 7 10.1186/1746-4269-7-9 PMC3060860 21385357

[B432] Coimbra CEA Jr Estudos de ecologia humana entre os Suruí do Parque Indígena Aripuanã, Rondônia. Aspectos alimentares Boletim do Museu Paraense Emílio Goeldi 1985 2 57 87

[B433] Coimbra CEA Jr Estudos de ecologia humana entre os Suruí do Parque Indígena Aripuanã, Rondônia. Elementos de etnozoologia Boletim do Museu Paraense Emílio Goeldi 1985 2 9 36

[B434] Carvalho JCM Lima PE Galvão E Observações zoológicas e antropológicas na região dos formadores do Xingu Publicações Avulsas do Museu Nacional 1949 5 1 4921771129

[B435] Pezzuti JCB Chaves RP Etnografia e uso dos recursos naturais pelos Índios Deni, Amazonas, Brasil Acta Amazonica 2009 39 121 138 10.1590/S0044-59672009000100013

[B436] Silva AL Comida de gente: preferências e tabus alimentares entre os ribeirinhos do Médio Rio Negro (Amazonas, Brasil) Revista de Antropologia 2007 50 125 179 10.1590/S0034-77012007000100004

[B437] Silva AL Begossi A Biodiversity, food consumption and ecological niche dimension: a study case of the riverine populations from the Rio Negro, Amazonia, Brazil Environment, Development and Sustainability 2009 11 3 489 507 10.1007/s10668-007-9126-z

[B438] Smith NJ Spotted cats and the Amazon skin trade Orxy 1976 13 362 371 10.1017/S0030605300014095

[B439] Pezzuti J Chaves RP Etnografia e manejo de recursos naturais pelos índios Deni, Amazonas, Brasil Acta Amazonica 2009 39 121 138 10.1590/S0044-59672009000100013

[B440] Terra AK Rebêlo GH Nelson E, Marques F, Vizoni V, Melo S O uso da fauna pelos moradores da Comunidade São João e Colônia Central Biotupé: Meio Físico, Diversidade Biológica e Sociocultural do Baixo Rio Negro, Amazônia Central 2005 1 Manaus, Brazil: INPA 141 153

[B441] Bandeira FPSFC Etnobiologia Pankararé 1993 1 Salvador, Bahia: Instituto de Biologia, Universidade Federal da Bahia

[B442] Lima KEC Vasconcelos SD Acidentes com animais peçonhentos: um estudo etnozoológico com agricultores de Tacaratu, sertão de Pernambuco Sitientibus 2006 6 138 144

[B443] Silva TS Freire EMX Freire EMX Fauna e Flora da Estação Ecológica do Seridó, Rio Grande do Norte: percepções e usos pelas comunidades do seu entorno Recursos Naturais das Caatingas: uma visão multidisciplinar 2009 1 Natal, RN, Brazil: UFRN 85 129

[B444] Hoefle SW O Sertanejo e os Bichos: Cognição Ambiental na Zona Semi-Árida Nordestina Revista da Antropologia 1990 33 47 74

[B445] Carvalho JCM Relações entre os índios do Alto Xingu ea fauna regional Publicações avulsas do Museu Nacional 1951 721771129

[B446] Setz EZF Animals in the Nambiquara diet: Methods of collection and processing Journal of Ethnobiology 1991 11 1 22

[B447] Thé APG Nordi N Common Property Resource System in a Fishery of the San Francisco River, Minas Gerais, Brazil Human Ecology Review 2006 13 1 10

[B448] Begossi A Madi E Fonseca M Castelo Branco P Silvano RAM Hogan D Pesca e consumo de pescado: uso de recursos por populações ribeirinhas do Piracicaba Caderno 2 - Qualidade Ambiental e Desenvolvimento regional nas bacias dos Rios Piacicaba e Capivari 1998 São Paulo: UNICAMP

[B449] MacCord PF Begossi A Dietary changes over time in a caiçara community from the Brazilian Atlantic Forest Ecology and Society 2006 11 38

[B450] Castro F Begossi A Ecology of fishing on the Grande River (Brazil): technology and territorial rights Fisheries Research 1995 23 361 373 10.1016/0165-7836(94)00343-U

[B451] Castro F Begossi A Fishing at Rio Grande (Brazil): ecological niche and competition Human Ecology 1996 24 401 411 10.1007/BF02169397

[B452] Torres DF Oliveira ES Alves RRN Vasconcellos A Etnobotânica e Etnozoologia em Unidades de Conservação: Uso da biodiversidade na Apa de Genipabu, Rio Grande do Norte, Brasil Interciencia 2009 34 623 629

[B453] Oliveira ES Torres DdF Alves RRN Vasconcellos A Alves RRN, Souto WMS, Mourão JS Etnozoologia em áreas protegidas: uso da fauna por populações locais na APA Bonfim/Guaraíras, Rio Grande do Norte, Brasil A Etnozoologia no Brasil: Importância, Status atual e Perspectivas 2010 7 1 Recife, PE, Brazil: NUPEEA 403 42221461541

[B454] Alves MS Silva MA Júnior MM Paranaguá MN Pinto SL Zooartesanato comercializado em Recife, Pernambuco, Brasil Revista Brasileira de Zoociências 2006 8 99 10921755223

[B455] Alarcon DT Schiavetti A O conhecimento dos pescadores artesanais de Itacaré sobre a fauna de vertebrados (não peixes) associados às atividades pesqueiras Gerenciamento Costeiro Integrado 2005 4 1 4

[B456] Alves RRN Nishida AK Aspectos socioeconômicos e percepção ambiental dos catadores de caranguejo-uçá *Ucides cordatus cordatus *(L. 1763) (Decapoda, Brachyura) do estuário do Rio Mamanguape, Nordeste do Brasil Interciencia 2003 28 36 43

[B457] Alves R Nishida A Hernandez M Environmental perception of gatherers of the crab 'caranguejo-uca' (Ucides cordatus, Decapoda, Brachyura) affecting their collection attitudes Journal of Ethnobiology and Ethnomedicine 2005 1 10 10.1186/1746-4269-1-10 16270913 PMC1289291

[B458] Begossi A The fishers and buyers from Buzios Island (Brazil): Kin ties and production Ciência e Cultura (SBPC) 1996 48 142 14721774139

[B459] Begossi A Richerson PJ The animal diet of families from Búzios island (Brazil): An optimal foraging approach Journal of Human Ecology 1992 3 433 458

[B460] Costa-Neto EM Restrições e preferências alimentares em comunidades de pescadores do município do Conde, Estado da Bahia, Brasil Revista de Nutrição 2000 13 117 126

[B461] Nishida AK Nordi N Alves RRN Aspectos socioeconômicos dos catadores de moluscos do litoral paraibano, Nordeste do Brasil Revista de Biologia e Ciências da Terra 2008 8 207 215

[B462] Nishida AK Nordi N Alves RRN Embarcações utilizadas por pescadores estuarinos da Paraíba, Nordeste Brasil Revista de Biologia e Farmácia 2008 3 1 8

[B463] Souto FJB Kubo RR, Bassi JB, Souza GC, Alencar NL, Medeiros PM, Albuquerque UP Sociobiodiversidade na pesca artesanal do litoral da Bahia Atualidades em Etnobiologia e Etnoecologia 2006 3 Recife, PE, Brazil: Livro Rápido 259 274

[B464] Docio L Razera JCC Pinheiro US Representações sociais dos moradores da Baía de Camamu sobre o Filo Porifera Ciência & Educação 2009 15 613 62921774139

[B465] Nishida AK Nordi N Alves RRN The lunar-tide cycle viewed by crustacean and mollusc gatherers in the State of Paraíba, Northeast Brazil and their influence in collection attitudes Journal of Ethnobiology and Ethnomedicine 2006 2 1 12 10.1186/1746-4269-2-1 16393342 PMC1360678

[B466] Monteles JS Castro TCS Viana DCP Conceição FS França VL Funo ICSA Percepção socio-ambiental das marisqueiras no município de Raposa, Maranhão, Brasil Revista Brasileira de Engenharia de Pesca 2009 4 34 45

[B467] Silva GS Mello RL Nascimento AE Messias A Araújo SFS A sustentabilidade ecológica das atividades pesqueiras artesanais e a relação com a malacofauna no manguezal do Rio Formoso-PE-Brasil Trabalhos Oceanográficos 2000 28 195 207

[B468] Botelho ERO Santos MCF Cata de crustáceos e moluscos no manguezal do Rio Camaragibe - Estado de Alagoas: Aspectos Sócio-ambiental e Técnico-econômico Boletim Técnico Científico da CEPENE 2005 13 77 96

[B469] Clauzet M Ramires M Barella W Pesca Artesanal e conhecimento local de duas populações caiçaras (Enseada do mar virado e Barra Una) no litoral de São Paulo Multiciência 2005 4 1 22

[B470] Bahia NCF Bondioli ACV Interação das tartarugas marinhas com a pesca artesanal de cerco-fixo em Cananéia, litoral sul de São Paulo Revista Biotemas 2010 23 3 203 213

[B471] Docio L Tolentino-Lima MA Costa-Neto EM Jucá-Chagas R Pinheiro U Interações ecológicas de esponjas marinhas (Animalia, Porifera) segundo pescadores artesanais da Baía de Camamu, Bahia, Brasil Revista Biotemas 2010 23 3 181 189

[B472] Zappes CA Nery MF Andriolo A Simão SM Ethnobiology and photo-identification: identifying anthropic impacts on boto-cinza dolphin *Sotalia guianensis *in Sepetiba Bay, Brazil Revista Brasileira de Biociências 2010 8 2 221 224

[B473] Alves HN Nordi N Nishida AK Alves RRN Alves RRN, Souto WMS, Mourão JS Perfil nutricional de catadores de Caranguejo-uçá (*U. Cordatus*) do distrito de Várzea Nova, Santa Rita/PB A Etnozoologia no Brasil: Importância, Status atual e Perspectivas 2010 7 1 Recife, PE, Brazil: NUPEEA 251 27621461541

[B474] Alves MS Silva MA Pinto SL Perfil sócio-econômico dos atores envolvidos na produção e comercialização de zooartesanato em Recife, Pernambuco - Brasil Revista Nordestina de Zoologia 2010 4 97 104

[B475] Dias TLP Alves RRN Léo Neto NA Alves RRN, Souto WMS, Mourão JS Zooartesanato marinho da Paraíba A Etnozoologia no Brasil: Importância, Status atual e Perspectivas 2010 7 1 Recife, PE, Brazil: NUPEEA 513 53421461541

[B476] Pinto MF Silva JRF Alves RRN Nishida AK Alves RRN, Souto WMS, Mourão JS Os animais do manguezal do estuário do Rio Jaguaribe, Aracati, Ceará - Uma abordagem etnozoológica A Etnozoologia no Brasil: Importância, Status atual e Perspectivas 2010 7 1 Recife, PE, Brazil: NUPEEA 233 25021461541

[B477] Pieve SMN Kubo RR Coelho-de-Souza G Pescadores da Lagoa Mirim Etnoecologia e Resiliência 2009 1 Brasília, DF, Brazil: Ministério do Desenvolvimento Agrário do Brasil

[B478] Begossi A Hanazaki N Ramos RM Food Chain and the Reasons for Fish Food Taboos among Amazonian and Atlantic Forest Fishers (Brazil) Ecological Applications 2004 14 1334 1343 10.1890/03-5072

[B479] Magalhães A Costa RM Silva Rd Pereira LCC The role of women in the mangrove crab (Ucides cordatus, Ocypodidae) production process in North Brazil (Amazon region, Pará) Ecological Economics 2007 61 559 565 10.1016/j.ecolecon.2006.05.013

[B480] Ayres OM Os animais dos Campos Gerais (PR): Impactos ambientais noticiados pela imprensa regional Publicatio UEPG: Ciências Biológicas e da Saúde 2006 12 7 19

[B481] Baldus H Vocabulário zoológico Kaingang Arquivos do Museu Paranaense 1947 6 149 160

[B482] Bizerril MXA Andrade TCS Knowledge of the urban population about fauna: Comparison between Brazilian and exotic animals Ciencia e Cultura 1999 51 38 41

[B483] Câmara Cascudo L Tradições populares da pecuária nordestina 1956 1 Ministério da Agricultura, Serviço de Informação Agrícola17638258

[B484] Cavallini MM Nordi N Ecological niche of family farmers in southern Minas Gerais state (Brazil) Brazilian Journal of Biology 2005 65 61 66 10.1590/s1519-69842005000100009 16025904

[B485] Costa Neto EM Santos-Fita D Vargas-Clavijo M Manual de Etnozoología: Una guía teórico-práctica para investigar la interconexión del ser humano con los animales 2009 1 Valencia, Spain: Tundra Ediciones21768914

[B486] Costa-Neto EM Queiroz LPd, Rapini A, Giulietti AM Ethnozoology of the Semi-arid of Bahia: Study cases Towards greater knowledge of the Brazilian Semi-arid biodiversity 2006 Brasília, Brazil: Ministério da Ciência e Tecnologia 109 112

[B487] Gurgel-Gonçalves R Costa-Neto EM, Santos-Fita D, Clavijo MV Etnoparasitología Manual de Etnozoología: una guía teórica-práctica para investigar la interconexión del ser humano con los animales 2009 1 Valencia, Spain: Tundra Ediciones 176 19921768914

[B488] Jalles Filho E Perspectivas darwinistas no estudo de sociedades caçadoras e coletoras: etnografia e etnoarqueologia Anais de Etologia 1996 14 19 27

[B489] Luiz MSF Bertazzoni EC Vilas Boas JC Perrelli MAS Perrelli MAS, Albuquerque LB, Anjos-Aquino EA Contribuições à etnozoologia xavante: estudos dos artefatos expostos no Museu Dom Bosco, Campo Grande-MS Descobrindo o Museu Dom Bosco: Experiencias de pesquisa e extensão no Museu Dom Bosco 2005 1 Campo Grande, MS, Brazil: Editora UCDB 173 178

[B490] Marques JGW Pescando pescadores. Ciência e etnociência em uma perspectiva ecológica 2001 2 São Paulo, Brazil: NUPAUB

[B491] Marques JGW Pescando Pescadores: Etnoecologia abrangente no baixo São Francisco Alagoano 1995 1 São Paulo, Brazil: NUPAUB/USP

[B492] Paiva MP Campos E Fauna do nordeste do Brasil: conhecimento científico e popular 1995 1 Fortaleza, CE, Brazil: Banco do Nordeste

[B493] Vanzolini PE Notas sobre a Zoologia dos índios Canela Revista do Museu Paulista 1958 10 155 171

[B494] Alves RRN Silva CC Alves HN Aspectos sócio-econômicos do comércio de plantas e animais medicinais em área metropolitanas do Norte e Nordeste do Brasil Revista de Biologia e Ciências da Terra 2008 8 181 189

[B495] Nomura H Folclore dos animais inferiores 2003 Mossoró, RN: Fundação Vingt-un Rosado

[B496] Alves AGC Pires DAF Ribeiro MN Conhecimento local e produção animal: Uma perspectiva baseada na Etnozootecnia Arch Zootec 2010 59 45 56

[B497] Marques JGW Alves AGC Souto FJB Peroni N Alves AGC, Souto FJB, Peroni N O Camboeiro de Setembro e as Ladainhas de Maio: Comunidades Tradicionais Pesqueiras do Brasil e sua Inserção no Nicho Ecológico Etnoecologia em Perspectiva Natureza, Cultura e Conservação 2010 3 Recife, PE: NUPEEA 129 141

[B498] Alves RRN Souto WMS Alves RRN, Souto WMS, Mourão JS Panorama atual, avanços e perspectivas futuras para Etnozoologia no Brasil A Etnozoologia no Brasil: Importância, Status atual e Perspectivas 2010 7 1 Recife, PE, Brazil: NUPEEA 41 5621461541

[B499] Alves RRN Souto WMS Alves RRN, Souto WMS, Mourão JS Desafios e dificuldades associadas as pesquisas etnozoológicas no Brasil A Etnozoologia no Brasil: Importância, Status atual e Perspectivas 2010 7 1 Recife, PE, Brazil: NUPEEA 57 6621461541

[B500] Alves RRN Souto WMS Mourão JS A Etnozoologia no Brasil: Importância, Status atual e Perspectivas 2010 1 Recife, PE, Brazil: NUPEEA21461541

[B501] Ferreira FS Brito SV Fernandes-Ferreira H Alves RRN Costa-Neto EM, Alves RRN Prospecção biológica, recursos zooterápicos e sustentabilidade Zooterapia: Os Animais na Medicina Popular Brasileira 2010 2 1 Recife, PE, Brazil: NUPEEA 141 158

[B502] Lopez LCS Souto WMS Ferreira FS Alves RRN Alves RRN, Souto WMS, Mourão JS Uma perspectiva de ecologia de comunidades aplicada à análise de dados de etnozoologia A Etnozoologia no Brasil: Importância, Status atual e Perspectivas 2010 7 1 Recife, PE, Brazil: NUPEEA 535 55021461541

[B503] Nishida AK Nascimento RQ Pinto MF Menezes VC Maia GC Alves RRN, Souto WMS, Mourão JS Tecnologia rudimentar empregada no beneficiamento de mariscos no litoral paraibano versus pressão sobre o estoque pesqueiro A Etnozoologia no Brasil: Importância, Status atual e Perspectivas 2010 7 1 Recife, PE, Brazil: NUPEEA 177 19221461541

[B504] Santos LD Costa-Neto EM Fuentes AM, Silva MTP, Méndez RM, Azúa RV, Correa PM, Santillán TVG Registro dos usos culturais de esponjas (Animalia Porifera) no Brasil e no mundo Sistemas biocognitivos tradicionales: Paradigmas en la conservación biológica y el fortalecimiento cultural 2010 1 Pachuca, Mexico: Universidad Autónoma del Estado de Hidalgo, Asociación Etnobiológica Mexicana y Sociedad Latinoamericana de Etnobiología 18 23

[B505] Souza ACFF Vieira DM Teixeira SF Alves RRN, Souto WMS, Mourão JS Trabalhadores da Maré: Conhecimento tradicional dos pescadores de moluscos na área urbana de Recife-PE A Etnozoologia no Brasil: Importância, Status atual e Perspectivas 2010 7 1 Recife, PE, Brazil: NUPEEA 149 17621461541

[B506] Alves LCPS Andriolo A Caracterização preliminar do comércio ilegal de animais silvestres na Feira Livre do Bairro da Liberdade, Manacapuru, Estado do Pará Sitientibus 2010 10 236 243

